# Semiparametric efficient estimation of small genetic effects in large-scale population cohorts

**DOI:** 10.1093/biostatistics/kxaf030

**Published:** 2025-09-30

**Authors:** Olivier Labayle, Breeshey Roskams-Hieter, Joshua Slaughter, Kelsey Tetley-Campbell, Mark J van der Laan, Chris P Ponting, Sjoerd V Beentjes, Ava Khamseh

**Affiliations:** School of Informatics, University of Edinburgh, 10 Crichton Street, Edinburgh EH8 9AB, United Kingdom; Institute for Regeneration and Repair, University of Edinburgh, 4–5 Little France Drive, Edinburgh EH16 4UU, United Kingdom; Health Data Research UK, 215 Euston Road, London NW1 2BE, United Kingdom; MRC Human Genetics Unit, Institute of Genetics and Cancer, University of Edinburgh, Crewe Road South, Edinburgh EH4 2XU, United Kingdom; School of Informatics, University of Edinburgh, 10 Crichton Street, Edinburgh EH8 9AB, United Kingdom; MRC Human Genetics Unit, Institute of Genetics and Cancer, University of Edinburgh, Crewe Road South, Edinburgh EH4 2XU, United Kingdom; School of Public Health, University of California, Berkeley, 2121 Berkeley Way, Berkeley, CA 94720, United States; MRC Human Genetics Unit, Institute of Genetics and Cancer, University of Edinburgh, Crewe Road South, Edinburgh EH4 2XU, United Kingdom; MRC Human Genetics Unit, Institute of Genetics and Cancer, University of Edinburgh, Crewe Road South, Edinburgh EH4 2XU, United Kingdom; School of Public Health, University of California, Berkeley, 2121 Berkeley Way, Berkeley, CA 94720, United States; School of Mathematics and Maxwell Institute for Mathematical Sciences, University of Edinburgh, Peter Guthrie Tait Road, Edinburgh EH9 3FD, United Kingdom; School of Informatics, University of Edinburgh, 10 Crichton Street, Edinburgh EH8 9AB, United Kingdom; MRC Human Genetics Unit, Institute of Genetics and Cancer, University of Edinburgh, Crewe Road South, Edinburgh EH4 2XU, United Kingdom; School of Public Health, University of California, Berkeley, 2121 Berkeley Way, Berkeley, CA 94720, United States

**Keywords:** non-parametric methods, bioinformatics, statistical genetics, statistical methods in epidemiology

## Abstract

Population genetics seeks to quantify DNA variant associations with traits or diseases, as well as interactions among variants and with environmental factors. Computing millions of estimates in large cohorts in which small effect sizes and tight confidence intervals are expected, necessitates minimizing model-misspecification bias to increase power and control false discoveries. We present TarGene, a unified statistical workflow for the semi-parametric efficient and double robust estimation of genetic effects including $ k $-point interactions among categorical variables in the presence of confounding and weak population dependence. $ k $-point interactions, or Average Interaction Effects (AIEs), are a direct generalization of the usual average treatment effect (ATE). We estimate genetic effects with cross-validated and/or weighted versions of Targeted Minimum Loss-based Estimators (TMLE) and One-Step Estimators (OSE). The effect of dependence among data units on variance estimates is corrected by using sieve plateau variance estimators based on genetic relatedness across the units. We present extensive realistic simulations to demonstrate power, coverage, and control of type I error. Our motivating application is the targeted estimation of genetic effects on trait, including two-point and higher-order gene-gene and gene-environment interactions, in large-scale genomic databases such as UK Biobank and *All of Us*. All cross-validated and/or weighted TMLE and OSE for the AIE $ k $-point interaction, as well as ATEs, conditional ATEs and functions thereof, are implemented in the general purpose Julia package TMLE.jl. For high-throughput applications in population genomics, we provide the open-source Nextflow pipeline and software TarGene which integrates seamlessly with modern high-performance and cloud computing platforms.

## 1. INTRODUCTION

Over the past 15 yrs, many genetic risk loci have been associated with disease or traits (Visscher et al. 2017). Most genetic effect sizes are small and require large samples to be reliably estimated (Uffelmann et al. 2021). Their detection helps guide interventions on the downstream disease consequences of the variant that may be both sizeable and clinically relevant (King et al. 2019; Minikel et al. 2024). Nevertheless, a slight bias, such as when an estimation model is misspecified, can appreciably alter genetic effect estimates and, additionally, their associated confidence intervals may not have the theoretical nominal coverage of the ground truth. This problem of bias is exacerbated for larger sample sizes that yield more confident estimates. Statistical models in common use, such as logistic, linear and linear mixed models (LMMs) (Uffelmann et al. 2021), rely on parametric assumptions, including linearity of the variant-covariate-trait relationship, and normality of the associated conditional distribution. LMMs also usually include a random effect to capture population relatedness (Lippert et al. 2011; Svishcheva et al. 2012; Uffelmann et al. 2021).

When genotype assignment is entirely random (ie without population stratification), and when non-linear genotype effects are absent, the commonly used LMM model is adequate for estimating the true effect size consistently. Nevertheless, genetic effects are often non-linear (Palmer et al. 2023; Mackay and Anholt 2024; Milind et al. 2024), and have been accounted for as an additive-plus-dominance part of an LMM (Huang and Mackay 2016). Also, warped LMM has generalized LMMs by transforming outcomes to account for non-Gaussian distributed residuals (Fusi et al. 2014). Recent applications of ML algorithms, such as DeepNull (McCaw et al. 2022), can adaptively account for covariate non-linearities using neural networks or XGBoost, yet still assume linearity of genetic effects for statistical estimation. Furthermore, for any variant and trait combination, the validity of any linearity assumption is a priori unknown, and so provides no guarantee that the LMM is a consistent estimator with nominal coverage.

Beyond single variant associations, interactions among multiple variants, known as epistasis, are more complicated to estimate (Wei et al. 2014) and may be smaller yet are crucial to understanding complex disease (Mackay and Anholt 2024). Robust estimation techniques are needed to accurately estimate genetic effects, together with interactions among variants, and interactions among variants and environmental factors. Targeted Learning (TL) offers a step-by-step framework for estimating causal and statistical parameters from real-world data with minimal bias (Gruber et al. 2024). This includes the construction of semi-parametric efficient substitution estimators, including Targeted Maximum Likelihood Estimators (TMLE), under minimal assumptions (van der Laan and Rubin 2006). TL has been successfully applied in clinical and biomedical settings to generate real-world evidence (Havlir et al. 2019; Fong et al. 2022; Gilbert et al. 2022).

Here, we introduce Targeted Genomic Estimation (TarGene), a method based on targeted semi-parametric estimation theory (Pfanzagl and Wefelmeyer 1985; van der Laan and Rose 2011) applying the TL roadmap to the estimation of genetic effects of single variants and interactions. The five key contributions of this paper are as follows. (i) Development of semi-parametric estimators, namely, Targeted Maximum Likelihood Estimator (TMLE), weighted TMLE (wTMLE), one-step estimator (OSE) and their cross-validated (CV) versions, for $ k $-point interaction effects on outcome, here among genetic variants, and genetic variants and environmental factors. (ii) Accounting for population dependence structure, by updating variance estimates via a network adaptation of the sieve plateau variance estimator of Davies and van der Laan (2014). (iii) Extensive simulations to assess type I error, power, bias, and coverage of various semi-parametric estimators and target parameters in realistic population genetics contexts, including (rare) binary outcomes, continuous outcomes, and categorical variants with diverse population frequencies. (iv) A general purpose Julia software package, TMLE.jl (Labayle et al., 2025), implements these semi-parametric estimators of our $ k $-point interaction parameters. (v) An end-to-end Nextflow pipeline, software and documentation, TarGene, for scalable and seamless application to large-scale biobanks (0.5 million or more individuals) such as the UK Biobank (Bycroft et al. 2018) and the *All of Us* cohort (Bick et al. 2024).

As an illustration, we apply TarGene to five real-data settings in UK Biobank. First, we perform a Phenome-wide Association Study (PheWAS) for the *FTO* intronic variant rs1421085, a candidate causal variant for obesity (Claussnitzer et al. 2015) on 768 traits, including body mass index (BMI). We find that *P*-values currently reported in the literature may be inflated thereby leading to a higher false discovery rate. Second, we simultaneously discover non-linear effect sizes of additional allelic copies on trait or disease. Specifically, we demonstrate significant genetic non-linearity at the *FTO* locus for 39 traits in this study. Third, for the same locus, we find evidence of gene-by-environment interactions (G$ \times $E) using deprivation indices on body weight-related traits. Fourth, we further reproduce five pairs of epistatic loci associated with red hair and find 16 further epistatic interactions on hair or skin color (G$ \times $G). Fifth, we illustrate how TarGene can be used to investigate higher-order interactions (G$ \times $G$ \times $G) using three variants linked to the vitamin D receptor complex.

This paper is organized as follows. In Section 2, we introduce the k-point interaction target parameter, derive its efficient influence function, and exact second-order remainder. This allows for the construction of various semi-parametric efficient estimators and their cross-validated analogues. We discuss various hypothesis testing strategies, and adapt sieve variance plateau estimators to account for population dependence in a network setting. In Section 3, we describe two extensive simulations: A null simulation of no genetic effect and a realistic simulation modeled on real UK Biobank data. We assess our estimators’ performance in terms of power and coverage. Subsequently, we apply TarGene to perform three population genetics analyses on UK Biobank (UKB) data: a PheWAS, a $ G\times E $ interaction study, and a third-order $ G\times G\times G $ study in Section 4. We also compare the genetic effect of the FTO variant on BMI between UKB and the *All of Us* cohort. Finally, Section 5 provides details of our Julia package TMLE.jl, Nextflow pipeline TarGene, and runtime considerations.

## 2. EFFICIENT ESTIMATION OF *K*-POINT INTERACTIONS

### 2.1. Inferential problem

Consider the observed data unit $ O=(W, A_{1},A_{2},\ldots, A_{m},Y)\sim P_{0} $, where $ W $ is a vector of pre-treatment covariates, the $ A_{i}\in\{0,1, \ldots t_{i}\} $ with $ i\,=\,1, \ldots, m $ are categorical treatment variables, $ Y $ is an outcome of interest, and $ P_{0}\in{\mathcal{M}} $ is the true data-generating probability distribution. In genomics, $ Y $ denotes a disease or trait, the $ A_{i} $ encode DNA variants at particular locations in the genome, and $ W $ is a vector of genetic confounders due to ancestry and relatedness, usually captured by principal components. Since we do not wish to impose any potentially unrealistic assumptions on the statistical model $ {\mathcal{M}} $—and hence $ P_{0} $—we take $ {\mathcal{M}}={\mathcal{M}}_{0} $, the non-parametric model. However, our derivations carry through under arbitrary restrictions on the distributions of the $ A_{i} $ given $ W $.

Our statistical estimand of interest is the $ k $-point interaction among a subset of the treatment variables $ A_{1},\ldots, A_{m} $ in their effect on outcome $ Y $ whilst correcting for covariates $ W $. This parameter is a generalized difference of mean outcomes, and best illustrated by an example. Let $ \bar{Q}_{a_{1},a_{2}}(w)=\bar{Q}(a_{1},a_{2},w)={\mathbb{E}}_{P}(Y|A_{1}=a_{1},A_{2}=a_{2},W\,=\,w) $ denote the outcome regression, and let $ Q_{W}(w)=P(W\leq w) $ denote the cumulative distribution function of the covariates $ W $. The 2-point interaction parameter $ \Psi_{a(0),a(1)}^{(2)}\colon{\mathcal{M}}_{0}\to{\mathbb{R}} $ of $ A_{1} $ and $ A_{2} $ in their effect on $ Y $ is defined as

1 \begin{align*}\Psi_{a(0),a(1)}^{(2)}(P)=\int\left[\left\{\bar{Q}_{1,1}(w)-\bar{Q}_{0,1}(w)\right\}-\left\{\bar{Q}_{1,0}(w)-\bar{Q}_{0,0}(w)\right\}\right]dQ_{W}(w),\end{align*}

where $ P\in{\mathcal{M}}_{0} $ and the treatment levels change from baseline levels $ a(0)=(0,0) $ to target levels $ a(1)=(1,1) $ for $ A_{1},A_{2} $. The parameter $ \Psi_{a(0),a(1)}^{(2)}(P) $ quantifies the difference between the treatment effect of $ A_{1}\colon 0\to 1 $ on $ Y $ given $ A_{2}=1 $ and the treatment effect of $ A_{1}\colon 0\to 1 $ on $ Y $ given $ A_{2}=0 $, correcting for $ W $. Symmetrically, the parameter is the difference between the treatment effect of $ A_{2}\colon 0\to 1 $ on $ Y $ given $ A_{1}=1 $ and the treatment effect of $ A_{2}\colon 0\to 1 $ on $ Y $ given $ A_{1}=0 $, correcting for the marginal distribution of $ W $. It also represents the non-additive effect on $ Y $ due to the joint change in the treatment levels of $ A_{1} $ and $ A_{2} $ simultaneously, rather than individually (Hernan and Robins 2023). This can be seen from the following alternative presentation:

2 \begin{align*}\Psi_{a(0),a(1)}^{(2)}(P) & = \int \big[\{\bar{Q}_{1,1}(w)-\bar{Q}_{0,0}(w)\}-(\{\bar{Q}_{1,0}(w)-\bar{Q}_{0,0}(w)\}\nonumber\\
&\quad +\{\bar{Q}_{0,1}(w)-\bar{Q}_{0,0}(w)\})]dQ_{W}(w),\end{align*}

which follows by adding and subtracting the term $ \bar{Q}_{0,0}(w) $ from the integrand. Two-point interactions have previously been studied in the experimental design and causal inference literature (Cox 1984; VanderWeele and Knol 2014; Dasgupta et al. 2015; Egami and Imai 2019).

Example 1. *Let $ A_{1},A_{2} $ be binary treatment variables and consider a linear model with interaction term in which the coefficients can depend on the covariates $ W $, namely*
 

\begin{align*} Y=\alpha(W)+\beta_{1}(W)A_{1}+\beta_{2}(W)A_{2}+\gamma(W)A_{1}A_{2}+\epsilon, \qquad\epsilon\sim N(0, \sigma^{2}),\end{align*}

 *where $ {\mathbb{E}}[\epsilon|W]=0 $. The conditional mean $ \bar{Q}_{A_{1},A_{2}}(W)={\mathbb{E}}(Y|A_{1},A_{2},W) $ is the same linear expression with interaction term without the noise term $ \epsilon $. Evaluating the target parameter of (2) yields*
 

\begin{align*}
\Psi_{a(0),a(1)}^{(2)}(P) & =\int\big\{(\beta_{1}(W)+\beta_{2}(W)+\gamma(W))\\
&\quad{} -\left(\beta_{1}(W)+\beta_{2}(W)\right)\big\} dQ_{W}(w)= {\mathbb{E}}_{P}\{\gamma(W)\}.\end{align*}

*When $ \gamma(W)=\gamma $ is independent of covariates, this reduces to $ \Psi_{a(0),a(1)}^{(2)}(P)=\gamma $ which coincides with the usual notion of 2-point interaction in a linear model.*

Next, we give the general definition of the $ k $-point interaction among a subset of $ m $ treatment variables in their effect on outcome $ Y $ whilst correcting for covariates $ W $. Select $ k $ of the $ m $ treatment variables all pairwise distinct. For ease of exposition, and without loss of generality, we relabel these as the first $ k $ variables, ie $ A=(A_{1},\ldots, A_{k}) $. Let $ s\in\{0,1\}^{k} $ a binary vector of length $ k $, and let $ a(s)=(a_{1}(s_{1}),\ldots, a_{k}(s_{k})) $ indicate the joint specification of the $ k $ treatment levels. The joint baseline treatment level is denoted $ a(0)=(a_{1}(0),\ldots, a_{k}(0)) $, and the joint target treatment level $ a(1)=(a_{1}(1),\ldots, a_{k}(1)) $ where $ a_{i}(s)\in\{0,1, \ldots, t_{i}\} $. The $ k $-point interaction generalizes (2) and quantifies the non-additive effect due to the joint change in treatment levels of the variables $ A $ from $ a(0) $ to $ a(1) $ relative to the sum of the effects of marginal changes for all subsets of variables with the remaining treatment variables held at their initial level. Notationally, it is more convenient to consider the generalization of (1), defined in Beentjes and Khamseh (2020):

3 \begin{align*}\Psi^{(k)}_{a(0),a(1)}(P)=\sum\limits_{s\in\{0,1\}^{k}}(-1)^{k-(s_{1}+\ldots+s_{k})}\Psi_{a(s)}(P),\end{align*}

where $ \Psi_{a(s)}(P) $ is the treatment-specific, covariate-adjusted outcome mean defined by

4 \begin{align*}\Psi_{a(s)}(P) & ={\mathbb{E}}_{P}[{\mathbb{E}}_{P}(Y|A=a(s),W)]\nonumber\\& \equiv{\mathbb{E}}_{P}[{\mathbb{E}}_{P}(Y|A_{1}=a_{1}(s_{1}),\ldots, A_{k}=a_{k}(s_{k}),W)].\end{align*}

Under additional causal assumptions $ \Psi_{a(s)}(P) $ can be interpreted as the causal mean counterfactual outcome $ {\mathbb{E}}[Y(a(s))] $ under the joint treatment assignment $ A\,=\,a(s) $ where $ s\in\{0,1\}^{k} $ (Rubin 1974).

Before proceeding with the analysis of this target parameter, we recall some notation. As the parameter depends only on $ P $ through $ Q\,=\,Q(P)=(\bar{Q},Q_{W}) $, we sometimes write $ \Psi(Q) $ instead of $ \Psi(P) $. We denote the true outcome regression of $ Y $ on $ (A, W) $ by $ \bar{Q}_{0} $, the true covariate distribution by $ Q_{W, 0} $, and abbreviate both by $ Q(P_{0})=Q_{0}=(\bar{Q}_{0},Q_{W, 0}) $. We denote the propensity score by

5 \begin{align*} g(a(s),w)=P(A=a(s)\mid W=w)=P(A_{1}=a_{1}(s_{1}),\ldots, A_{k}=a_{k}(s_{k})\mid W =w).\end{align*}

Throughout, we assume the true propensity score $ g_{0} $ satisfies the positivity condition $ \delta < g_{0}(a(s),w) < 1-\delta $ for some $ \delta > 0 $, all covariates $ w $ in the support of $ Q_{W, 0} $, and all treatment assignments. Given a probability distribution $ P\in{\mathcal{M}}_{0} $ and any $ P $-integrable function $ f $, we write $ Pf={\mathbb{E}}_{P}[f(O)]=\int f(o)dP(o) $. The empirical distribution on $ n $ variables $ O_{1},\ldots, O_{n} $ is denoted by $ {\mathbb{P}}_{n} $, and the sample average of $ f $ with respect to $ {\mathbb{P}}_{n} $ by $ {\mathbb{P}}_{n}f=\frac{1}{n}\sum_{i\,=\,1}^{n}f(O_{i}) $. Henceforth, we leave the interaction order, as well as the initial and final treatment levels, implicit and write $ \Psi(P) $ for the $ k $-order interaction from levels $ a(0) $ to $ a(1) $ as defined in (3).

### 2.2. Semi-parametric efficient estimators

Let $ \hat{P}_{n} $ be an estimator of $ P_{0} $ based on the available data $ {\mathbb{P}}_{n} $, and denote by $ \hat{\psi}_{n}\equiv\Psi(\hat{P}_{n}) $ the corresponding plug-in estimator. Since $ \Psi $ is path-wise differentiable, it admits a Von Mises expansion:

6 \begin{align*}\Psi(\hat{P}_{n})-\Psi(P_{0})=(\hat{P}_{n}-P_{0})D^{*}(\hat{P}_{n})+R(\hat{P}_{n},P_{0}).\end{align*}

Here $ D^{*}(Q, g) $ is the efficient influence function (EIF) and the canonical gradient of the parameter and $ R(\hat{P}_{n},P_{0}) $ is the second-order exact remainder. The EIF is a key object in semi-parametric efficient estimation, as it characterizes the asymptotic behavior of any regular and efficient estimator (Bickel et al. 1998). The EIF of $ \Psi_{a}(P) $ at $ P $ relative to $ {\mathcal{M}}_{0} $ is

7 \begin{align*} D_{a}^{*}(P)(O)=D_{a}^{*}(Q, g)(O)=\frac{\mathbb{1}\{A=a\}}{g(A, W)}\left\{Y-\bar{Q}(A, W)\right\}+\bar{Q}(a, W)-\Psi_{a}(Q),\end{align*}

where $ O $ is distributed according to $ P\in{\mathcal{M}}_{0} $ (van der Laan and Robins 2003). Note that $ A\,=\,a $ can denote a treatment level of a single variable, or the joint treatment level specification of $ k $ treatments $ A=(A_{1},\ldots, A_{k}) $ (Supplementary Material). The second-order exact remainder of $ \Psi_{a}(P) $ at $ P $ relative to $ {\mathcal{M}}_{0} $ can be written as

8 \begin{align*} R_{a}(P, P_{0})=R_{a}\left(Q_{a},Q_{a, 0},g_{a},g_{a, 0}\right)=P_{0}\left\{\left(\bar{Q}_{a}-\bar{Q}_{a, 0}\right)\left(g_{a}-g_{a, 0}\right)/g_{a}\right\}\end{align*}

where we write $ \bar{Q}_{a}(W)=\bar{Q}(a, W) $, $ g_{a}(W)=P(A\,=\,a|W) $ and their analogues implied by $ P_{0} $ (van der Laan and Robins 2003). Since the $ k $-point interaction parameter is a linear combination of parameters $ \Psi_{a(s)}(P) $ as in (4), its EIF and second-order remainder are linear combinations of the EIF and second-order remainder of $ \Psi_{a(s)}(P) $ respectively. We formally establish the following result in the Supplementary Materials.

Proposition 1. The canonical gradient (or EIF) and second-order exact remainder of the $ k $-point interaction target parameter $ \Psi^{(k)}_{a(0),a(1)}(P) $ of (3) at $ P $ relative to the non-parametric model $ {\mathcal{M}}_{0} $ are equal to

9 \begin{align*} D^{*}_{a(0),a(1)}(P)=\sum\limits_{s\in\{0,1\}^{k}}(-1)^{k-(s_{1}+\ldots+s_{k})}D_{a(s)}^{*}(P)\end{align*}

10 \begin{align*} R_{a(0),a(1)}(P, P_{0})=\sum\limits_{s\in\{0,1\}^{k}}(-1)^{k-(s_{1}+\ldots+s_{k})}R_{a(s)}(P, P_ {0}),\end{align*}

respectively, where the expressions of $ D^{*}_{a(s)}(P) $ and $ R_{a(s)}(P, P_{0}) $ are given in (7) and (8).

Following Benkeser et al. (2017), the Von Mises expansion in (6) can be written as

11 \begin{align*}\Psi(Q_{n})-\Psi(Q_{0})=({\mathbb{P}}_{n}-P_{0})D^{*}(Q, g)-B_{n}(Q_{n}, g_{n})+M_{n}(Q_{n},Q, g_{n},g)+R(Q_{n},Q_{0},g_{n},g_{0})\end{align*}

where $ \bar{Q}_{n} $ and $ g_{n} $ are the relevant components of $ \hat{P}_{n} $, $ \bar{Q} $ and $ g $ their respective in-probability limits, and $ \bar{Q}_{0} $ and $ g_{0} $ the components of $ P_{0} $. In this decomposition, $ B_{n}(Q_{n},g_{n})={\mathbb{P}}_{n}D^{*}(Q_{n},g_{n}) $ is the first-order bias term and $ M_{n}(Q_{n},Q, g_{n},g)=({\mathbb{P}}_{n}-P_{0})\left\{D^{*}(Q_{n},g_{n})-D^{*}(Q, g)\right\} $ is the empirical process term. We use this decomposition to construct non-parametric efficient asymptotically linear estimators of interaction via (i) one-step bias correction (Pfanzagl and Wefelmeyer 1985), and (ii) targeted minimum loss-based estimation (TMLE) (van der Laan and Rubin 2006; van der Laan and Rose 2011, 2018).

The empirical process term is $ o_{P}(n^{-1/2}) $ under mild conditions, see Benkeser et al. (2017); Van der Vaart and Wellner (1996). The rate of the second-order exact remainder term is determined by how fast the nuisance functions $ \bar{Q}_{0} $ and $ g_{0} $ are estimated. Due to its double robust structure by (12) in the Supplementary Materials, this terms is also $ o_{P}(n^{-1/2}) $ provided the product of their rates is $ n^{-1/2} $. Sufficient rates of $ n^{-1/4} $ for estimating both $ \bar{Q}_{0} $ and $ g_{0} $ can be achieved by some algorithms, such as the Highly Adaptive Lasso (HAL) (Benkeser and van der Laan 2016). The first term, $ ({\mathbb{P}}_{n}-P_{0})D^{*}(Q, g) $, is the average of $ n $ independent and identically distributed copies of the random variable $ D^{*}(Q, g)(O)-P_{0}D^{*}(Q, g) $. This random variable has mean zero if either $ Q\,=\,Q_{0} $ or $ g\,=\,g_{0} $ (or both).

The first-order bias $ B_{n}(Q_{n},g_{n}) $ of the interaction parameter of (3) follows from (9):

12 \begin{align*} B_{n}(Q_{n},g_{n})=\frac{1}{n}\sum\limits_{i=1}^{n}\left[\sum\limits_{s\in\{0, 1\}^{k}}(-1)^{k-(s_{1}+\ldots+s_{k})}\frac{\mathbb{1}\{a_{i}=a(s)\}}{g_{n}(a_{i},w_{i})}\right]\left\{y_{i}-\bar{Q}_{n}(a_{i},w_{i})\right\},\end{align*}

where $ o_{i}=(y_{i},a_{i},w_{i})=(y_{i},a_{1, i},\ldots, a_{k, i},w_{i}) $ is the $ i $th observed data point. In practice, this term can be substantial. We discuss two general strategies to construct an asymptotically linear estimator of the $ k $-order interaction parameter that attain the non-parametric efficiency bound given by the variance of the EIF $ D^{*}(Q, g) $, provided the rate conditions for the empirical process term and second-order exact remainder are met. This is the case when $ D^{*}(Q_{n},g_{n}) $ belongs to a $ P_{0} $-Donsker class with probability tending to one and $ P_{0}\left\{D^{*}(Q_{n},g_{n})-D^{*}(Q, g)\right\}^{2} $ converges to zero in probability (Benkeser et al. 2017), or when we use sample splitting. Throughout, we refer to these as the *canonical* and the *cross-validated* (CV) approaches.

The One-Step Estimator (OSE) of Pfanzagl and Wefelmeyer (1985) is an infinite-dimensional generalization of the Newton-Raphson method in which the first-order bias term is added to the plug-in estimator:

13 \begin{align*}\hat{\psi}_{n}^{+}:=\hat{\psi}_{n}+B_{n}(Q_{n},g_{n}).\end{align*}

Under the rate conditions on $ M_{n} $ and $ R_{n} $, (11) reads $ \hat{\psi}^{+}_{n}-\psi_{0}={\mathbb{P}}_{n}D^{*}(Q, g)+o_{P}(n^{-1/2}) $, demonstrating that the OSE is asymptotically linear with variance equal to the non-parametric efficiency bound given by the variance of the EIF $ D^{*}(Q, g) $ provided $ Q\,=\,Q_{0} $ and $ g\,=\,g_{0} $. For the average treatment effect, ie our target parameter with $ k\,=\,1 $, the OSE reduces to the Augmented Inverse Propensity Weighting (AIPW) estimator introduced by Robins et al. (1994). While the OSE is asymptotically efficient and straightforward to implement, its finite-sample performance can suffer since it is not a plug-in estimator and the first-order bias term may push the estimate outside of the target parameter’s natural range.

The second approach, introduced by van der Laan and Rubin (2006), instead updates the fit $ \bar{Q}_{n} $ of $ \bar{Q}_{0} $ in an iterative procedure to a final fit $ \bar{Q}^{*}_{n} $ such that $ B_{n}(\bar{Q}^{*}_{n},g_{n})=0 $. This updating step yields the Targeted Maximum-Likelihood, or Targeted Minimum Loss-based, Estimator (TMLE),

14 \begin{align*}\hat{\psi}^{\textrm{tmle}}_{n}:=\Psi(Q^{*}_{n}).\end{align*}

Similar to OSE, (11) reads $ \hat{\psi}^{\textrm{tmle}}_{n}-\psi_{0}={\mathbb{P}}_{n}D^{*}(Q, g)+o_{P}(n^{-1/2}) $ under the rate conditions on $ M_{n} $ and $ R_{n} $, demonstrating that the TMLE is asymptotically linear with variance equal to the non-parametric efficiency bound given by the variance of the EIF $ D^{*}(Q, g) $ when $ Q\,=\,Q_{0} $ and $ g\,=\,g_{0} $. The sampling behavior of OSE and TMLE when either $ Q\,=\,Q_{0} $ or $ g\,=\,g_{0} $ is described in Section 2.2 of Benkeser et al. (2017). TMLE is a plug-in estimator and hence enjoys finite-sample robustness properties (Porter et al. 2011).

The TMLE updating step consists in a linear regression (for continuous outcome $ Y $) or logistic regression (for binary outcome $ Y $) with offset the initial fit $ \bar{Q}_{n}(a, w) $, and as single covariate the clever covariate $ H(g)(a, w) $. The clever covariate is derived from the EIF $ D^{*}(Q, g) $ and, for the $ k $-point interaction $ A\colon a(0)\to a(1) $, equals

15 \begin{align*} H(g_{n})(A, W)=\sum\limits_{s\in\{0,1\}^{k}}(-1)^{k-(s_{1}+\ldots+s_{k})}\frac{\mathbb{1}\{A=a(s)\}}{g_{n}(A, W)}\end{align*}

generalizing the clever covariate for the average treatment effect, ie interaction with $ k\,=\,1 $. We show in the Supplementary Materials that $ Q^{*}_{n}=(\bar{Q}^{*}_{n},\mathbb{Q}_{W}) $ solves the EIF, ie it eliminates the first-order bias $ B_{n}(Q^{*}_{n},g_{n})=0 $, where we use the empirical distribution $ \mathbb{Q}_{W} $ of $ Q_{W} $.

In finite samples, it has been observed in simulations (Sofrygin and van der Laan 2017) that performance in terms of bias, variance, and coverage improves when updating $ Q_{n} $ via weighted regression. Specifically, performance improves in the presence of near-positivity violations when the estimated propensity score $ g_{n}(A, W) $ is close to zero. This is particularly relevant in fields such as genetics, where DNA variants are frequently rare in a population. For this weighted approach, the term $ 1/g_{n}(A, W) $ is placed in the fluctuation loss function and removed from the clever covariate, which is then given by

16 \begin{align*} H^{\prime}(A, W)=\sum\limits_{s\in\{0,1\}^{k}}(-1)^{k-(s_{1}+\ldots+s_{k})}\mathbb{1}\{A=a(s)\}.\end{align*}

The weighted TMLE (wTMLE) is defined as $ \hat{\psi}_{n}^{\mathrm{wtmle}}\equiv\Psi\left(Q^{*}_{n}\right) $. We show in the Supplementary Material that the weighted fluctuations also solve the EIF, ie satisfy $ B_{n}(Q^{*}_{n},g_{n})=0 $.

Estimating the functions $ (Q_{n},g_{n}) $ with data-adaptive or ML algorithms whilst evaluating OSE and (w)TMLE on the same dataset may lead to decreased performance of the canonical estimators as it can affect the required rate conditions of the empirical process term $ M_{n} $. By using cross-validated (or sample-splitting) versions of these estimators, the $ P_{0} $-Donsker condition on algorithms used to fit $ Q_{n} $ and $ g_{n} $ can be avoided. Instead, we fit $ (Q_{n},g_{n}) $ on part of the data whilst maintaining performance of OSE and (w)TMLE by evaluating these estimators on a held-out part of the data, essentially treating the fits of $ (Q_{n},g_{n}) $ as fixed. We provide the definition of the CV-OSE (Kennedy et al. 2020), CV-TMLE (Zheng and van der Laan 2010) and its weighted version, CV-wTMLE, in the Supplementary Materials.

### 2.3. Inference and hypothesis testing

We obtain asymptotic Wald-type confidence intervals and hypothesis tests for interaction using the asymptotic normality of the interaction estimators constructed in the previous section. If $ \hat{\psi}_{n} $ is any of these estimators, with estimated nuisance functions $ (Q_{n},g_{n}) $, then

\begin{align*}\sqrt{n}\left(\hat{\psi}_{n}-\psi_{0}\right)=\sqrt{n}\,{\mathbb{P}}_{n}D^{*}(Q, g)+o_{P}(1)\rightsquigarrow\mathcal{N}\left(0,{\rm Var}D^{*}(Q, g)\right)\end{align*}

by the Central Limit Theorem. Here, recall that $ (Q, g) $ are the in-probability limits of $ (Q_{n},g_{n}) $ respectively, and we assume either $ Q\,=\,Q_{0} $ or $ g\,=\,g_{0} $ (or both). In practice, for the canonical estimators of (13) and (14), we use the sample variance estimator $ \hat{\sigma}_{n}^{2} $ built from $ (Q_{n},g_{n}) $ to approximate the variance of the EIF, namely

17 \begin{align*}\hat{\sigma}_{n}^{2}={\mathbb{P}}_{n}\left\{D^{*}(Q_{n},g_{n})\right\}^{2}=\frac{1}{n}\sum\limits_{i=1}^{n}\left\{D^{*}(Q_{n},g_{n})(o_{i})\right\}^{2}.\end{align*}

The cross-validated sample variance estimator $ \hat{\sigma}_{{\rm cv},n}^{2} $ of CV-OSE and CV-(w)TMLE can be found in the Supplementary Materials. Both $ \hat{\sigma}_{n}^{2} $ and $ \hat{\sigma}_{{\rm cv},n}^{2} $ are consistent estimators of $ {\rm Var}D^{*}(Q_{0},g_{0}) $ if the rate conditions on $ M_{n} $ and $ R_{n} $ hold. We obtain asymptotically valid $ (1-\alpha)\times 100\% $ Wald-type confidence intervals,

18 \begin{align*}\widehat{{\rm CI}}_{n}=\left(\hat{\psi}_{n}-z_{1-\alpha/2}\frac{\hat{\sigma}_{n}}{\sqrt{n}},\hat{\psi}_{n}+z_{1-\alpha/2}\frac{\hat{\sigma}_{n}}{\sqrt{n}}\right),\end{align*}

where $ z_{\beta} $ denotes the $ \beta $-quantile of the standard normal distribution. Similarly, under the null hypothesis of no interaction, $ H_{0}\colon\psi_{0}=0 $, we can use $ \sqrt{n}\hat{\psi}_{n}/\hat{\sigma}_{n}\sim{\mathcal{N}}(0,1) $ to test for interaction.

### 2.4. Estimands and estimators of interest in genetics

In a typical GWAS, the genetic effect size of a DNA variant on phenotype is estimated as a coefficient in a Linear Mixed Model (LMM). Due to the linearity assumption of the LMM, each DNA variant contributes a single test to any subsequent multiple testing procedure. In contrast, our first-order genetic effect size of (3) for $ k\,=\,1 $, estimates both allelic effects separately, yielding twice as many tests. For a $ k $-order interaction, this constitutes $ 2^{k} $ times as many tests. Instead, it is often of interest to understand whether a DNA variant at a locus, or a group of $ k $ DNA variants in an interaction, are significantly associated with a trait regardless of the specific genotype at the locus or loci. Joint testing is required because shared use of the heterozygote genotype at a locus makes the statistical tests dependent. Let $ \psi=(\psi_{1},\ldots, \psi_{p}) $ be a $ p $-dimensional estimand with canonical gradient $ D^{*}_{\psi}=(D^{*}_{\psi_{1}},\ldots, D^{*}_{\psi_{p}}) $. IF $ \hat{\psi}_{n}=(\hat{\psi}_{1, n},\ldots, \hat{\psi}_{p, n}) $ is a vector of asymptotically linear estimators with EIF $ D^{*}_{\psi} $, then

19 \begin{align*}\sqrt{n}\big(\hat{\psi}_{n}-\psi_{0}\big)=\sqrt{n}\,{\mathbb{P}}_{n}D^{*}(Q, g)+o_{P}(1)\rightsquigarrow\mathcal{N}\big(0,{\rm Var}D^{*}_{\psi}(Q, g)\big)\end{align*}

by the multi-variate Central Limit Theorem. Here $ \Sigma\equiv{\rm Var}D^{*}_{\psi}(Q, g) $ denotes the $ p\times p $-dimensional covariance matrix of $ D^{*}_{\psi}(Q, g) $ with in the $ jk $ entry the element $ \Sigma_{jk}={\rm Cov}\big(D^{*}_{\psi_{j}},D^{*}_{\psi_{k}}\big) $. In practice, we use the sample covariance matrix $ \hat{\Sigma}_{n} $ built from the estimated functions $ (Q_{n},g_{n}) $:

20 \begin{align*}\left(\hat{\Sigma}_{n}\right)_{jk}={\mathbb{P}}_{n}\left\{D^{*}_{\psi_{j}}(Q_{n},g_{n})D^{*}_{\psi_{k}}(Q_{n},g_{n})\right\}=\frac{1}{n}\sum\limits_{i=1}^{n}\left\{D^{*}_{\psi_{j}}(Q_{n},g_{n})(o_{i})\cdot D^{*}_{\psi_{k}}(Q_{n},g_{n})(o_{i})\right\}.\end{align*}

This is a consistent estimator of $ \Sigma $ provided the rate conditions on $ M_{n} $ and $ R_{n} $ hold for all $ \hat{\psi}_{i, n} $. To construct Wald-type confidence regions and test against the joint null $ H_{0}\colon\psi_{0}=0 $ that all $ p $ one-dimensional parameters are zero simultaneously, we use Hotelling’s $ t^{2}_{n}=n\left(\hat{\psi}_{n}-\psi_{0}\right)\hat{\Sigma}^{-1}_{n}\left(\hat{\psi}_{n}-\psi_{0}\right)^{T} $, where $ t_{n}^{2}\sim\frac{p(n-1)}{n-p}F_{p, n-p} $.

As an example, suppose $ k\,=\,1 $ and the treatment variable $ A\equiv A_{j_{1}} $ has three treatment levels denoted by $ \{0,1,2\} $. In genomics, $ \{0,1,2\} $ could represent the genotypes $ \{TT, TC, CC\} $ at a biallelic locus of the genome, where the treatment level corresponds to the number of copies of the minor allele $ C $. Consider the two linearly independent changes $ \psi_{1}\equiv\Psi_{0\to 1} $ and $ \psi_{2}\equiv\Psi_{1\to 2} $, corresponding to the changes $ a\colon 0\to 1 $ and $ a\colon 1\to 2 $ respectively. We seek to understand whether the addition of a single minor allele $ (0\to 1 $ or $ 1\to 2 $) has a significant effect on outcome $ Y $, correcting for covariates $ W $. The Hotelling statistic is $ t^{2}_{n}=n(\hat{\psi}_{1, n},\hat{\psi}_{2, n})\hat{\Sigma}^{-1}_{n}(\hat{\psi} _{1, n},\hat{\psi}_{2, n})^{T} $. Its corresponding confidence regions are ellipses with dimensions and orientation controlled by the constituent signal-to-noise ratios and the correlation of $ \hat{\psi}_{1, n} $ and $ \hat{\psi}_{2, n} $.

Another important question in genetics concerns the Allelic Effect Difference estimand $ \psi_{\Delta}=\psi_{2}-\psi_{1} $ contrasting the population effect of adding a second minor allele ($ 1\to 2 $) versus adding the first minor allele ($ 0\to 1 $). This estimator is obtained by applying the function $ f\colon{\mathbb{R}}^{2}\to{\mathbb{R}} $, $ f(x_{1},x_{2})=x_{2}-x_{1} $ to the estimand $ \psi=(\psi_{1},\psi_{2}) $. The functional delta method (Van der Vaart 2000) yields the asymptotic normality

21 \begin{align*}\sqrt{n}\left(\hat{\psi}_{\Delta, n}-\psi_{\Delta, 0}\right)\rightsquigarrow\mathcal{N}\left(0,{\rm Var}D^{*}_{\psi_{1}}-2{\rm Cov}(D^{*}_{\psi_{1}},D^{*}_{\psi_{2}})+{\rm Var}D^{*}_{\psi_{2}}\right),\end{align*}

incorporating the dependence of $ \hat{\psi}_{1, n} $ and $ \hat{\psi}_{2, n} $ via the covariance of their influence functions. The Allelic Effect Difference estimator $ \hat{\psi}_{\Delta} $ is directly available in the TMLE.jl package and TarGene software. Automatic differentiation functionalities in Julia allow the user to specify other functions of basic estimands.

### 2.5. Sieve Plateau variance estimators

Large-scale biobank cohorts consist of populations of participants who can be related due to genetic ancestry or kinship. Moreover, genetically similar individuals may share diet and environment inducing further dependence (Abdellaoui et al. 2022). This dependence among the variables $ O_{i} $ induces dependence among the influence functions of the estimands of interest. In TarGene, we account for the genetic dependence of individuals model-independently using an approach drawn from (Davies and van der Laan 2014). This approach generalizes the estimator of (17) for the variance of the $ k $-order interaction parameter, by constructing Sieve Plateau (SP) variance estimators as a function of the genetic similarity of individuals.

Genetic similarity of two individuals $ i $ and $ j $ is quantified by the sample correlation coefficient $ G_{ij} $ of a selection of their (zero-centred and scaled) DNA variants. Together, these coefficients form the Genetic Relationship Matrix (GRM), denoted $ G $, of size $ N\times N $ where $ N $ is the number of individuals in the population (Henderson 1975). More precisely, given a set of $ R $ variants, we have

22 \begin{align*} G_{ij}=\frac{1}{R-1}\sum\limits_{k=1}^{R}\frac{(s_{ik}-2p_{k})(s_{jk}-2p_{k})}{2p_{k} (1-p_{k})}.\end{align*}

Here $ s_{ik}\in\{0,1,2\} $ denotes the number of copies of the reference allele for individual $ i $ at variant $ k $, and $ p_{k}\in(0,1) $ denotes the frequency of the reference allele at variant $ k $ over the population of $ N $ individuals. In particular, we have $ \frac{1}{N}\sum_{i\,=\,1}^{N}s_{ik}=2p_{k} $. The $ R $ variants are typically chosen among uncorrelated, genotyped (not imputed) variants that approximately satisfy Hardy–Weinberg equilibrium, ie for which reference alleles follow a binomial distribution with mean frequency $ p_{k} $. The standard deviation of $ \tilde{s}_{ik} $ thus equals $ \sqrt{2p_{k}(1-p_{k})} $.

In general, the variance of the asymptotically normal OSE or (w)TMLE $ \hat{\psi}_{n} $ is given by

23 \begin{align*}\sigma_{n}^{2}={\rm Var}\left[{\mathbb{P}}_{n}D^{*}(Q, g)\right]=\frac{1}{n}\sum\limits_{i=1}^{n}\sum\limits_{j=1}^{n}{\rm Cov}\left(D^{*}(Q, g)(O_{i}),D^{*}(Q, g)(O_{j})\right).\end{align*}

Cross-covariance terms vanish if the EIFs of all participants are independent and, as before, we can use the sample variance estimator $ \hat{\sigma}_{n}^{2} $ built from the estimated functions $ (Q_{n},g_{n}) $ to estimate the variance of the EIF. The distinction between (17) and (23) is only relevant for sufficiently genetically similar individuals. SP variance estimators use a cut-off $ \tau $ for the genetic similarity between individuals, and set the covariance to zero if individuals are sufficiently genetically dissimilar. For a range $ \{\tau\in\mathrm{T}\} $, this produces a family of variance estimates, $ \{\hat{\sigma}_{n}^{2}(\tau):\tau\in\mathrm{T}\} $. Under appropriately weak dependence (see Davies and van der Laan (2014, Theorem 1)), the true variance is obtained where the function $ \tau\mapsto\hat{\sigma}_{n}^{2}(\tau) $ plateaus.

To construct SP variance estimators, we proceed as follows:

1. Using the GRM, we define genetic dissimilarity between individuals $ i $ and $ j $ as $ d(i, j)=1-G_{ij}\in[0,2] $. Biologically, if two individuals $ i $ and $ j $ have identical DNA variants, they are fully correlated, $ G_{ij}=+1 $, and thus have zero genetic distance, $ d(i, j)=0 $, as expected.
2. Given a value for the cut-off $ \tau\in[0,2] $, we define a SP variance estimator as
  24 \begin{align*}\hat{\sigma}^{2}_{n}(\tau)=\frac{1}{n}\sum\limits_{i=1}^{n}\sum\limits_{j=1}^{n}\mathbb{1}\{d(i, j)\leq\tau\}\cdot D^{*}(Q_{n},g_{n})(o_{i})D^{*}(Q_{n},g_{n})(o_{j}).\end{align*}
  The biological interpretation of these estimators is as follows. The correlation between the influence functions of individuals $ i $ and $ j $, estimated by the term $ D^{*}(Q_{n},g_{n})(o_{i})D^{*}(Q_{n},g_{n})(o_{j}) $, is included in the variance estimators only if the genetic dissimilarity between individuals $ i $ and $ j $ is at most $ \tau $. Thus, the SP variance estimator $ \hat{\sigma}^{2}_{n}(0) $ (for $ \tau\,=\,0 $) treats all individuals are *independent*. By increasing $ \tau $, we first take the covariance between strongly genetically dependent individuals into account for low $ \tau $, and then incorporate the covariance of more weakly dependent individuals as $ \tau $ increases to $ \tau\,=\,1 $.
3. We estimate $ \hat{\sigma}^{2}_{n}(\tau) $ for a range of values of the cut-off $ \tau $, *e.g.*, $ \tau=0, \frac{2}{k},\frac{4}{k},\ldots, \frac{2\cdot(k-1)}{k},2 $, and select the value $ \tau_{0} $ that maximizes this function, thereby yielding the most conservative variance estimate.
4. The selected Sieve Plateau variance estimate is $ \hat{\sigma}^{2}_{n}(\tau_{0}) $.

Under the assumptions of Davies and van der Laan (2014, Theorem 1), see also Davies and van der Laan (2014, Theorem 2), the estimator $ \hat{\sigma}^{2}_{n}(\tau_{0}) $ can be used instead of $ \hat{\sigma}^{2}_{n} $ in (18) to obtain confidence intervals and *P*-values accounting for population dependence.

## 3. SIMULATION STUDIES

While semi-parametric estimators are theoretically asymptotically optimal, asymptotic regimes may not be achieved in practice because events may be rare, even for large sample sizes. This is particularly prevalent in population genetics, where some genetic variants and traits are found in less than 1% of individuals. In the absence of finite sample guarantees, simulation studies provide an effective way to validate statistical methods. It has been recognised that simulations based on simple parametric models lack important features of the true generating process (Schuler et al. 2017; Li et al. 2022; Parikh et al. 2022) and, as a result, conclusions drawn from them may not generalize well to real-world data. More realistic simulations can be constructed by modeling the generating process with flexible generative models. While this approach does not yield a closed form expression of the ground truth, arbitrarily precise estimates can be obtained via Monte Carlo sampling.

Here we analyze the performance of our proposed semi-parametric estimators of $ k $-point interactions through two types of simulations. In both simulations, the set of confounders corresponds to the 6 first principal components (hereafter $ W=PCs $). A set of extra covariates influencing only the outcome $ Y $ is denoted by $ C $ and comprises at least age and genetic sex. Both simulations utilize the entire UK Biobank dataset, rather than a homogeneous subsample (*e.g.*, white British), and $ PCs $ and $ C $ are always sampled jointly to retain as many structural dependencies as possible. Finally, both simulations assume that interacting variants are not in linkage disequilibrium. That is, for two variants $ A_{1} $ and $ A_{2} $, $ \hat{P}_{n}(A_{1},A_{2}|PCs)=\hat{P}_{n}(A_{1}|PCs)\cdot\hat{P}_{n}(A_{2}|PCs) $. We demonstrate in a separate simulation (Supplementary Material S2.5) that linkage disequilibrium does not introduce additional estimation challenges beyond those presented here.

**Fig. 1.**
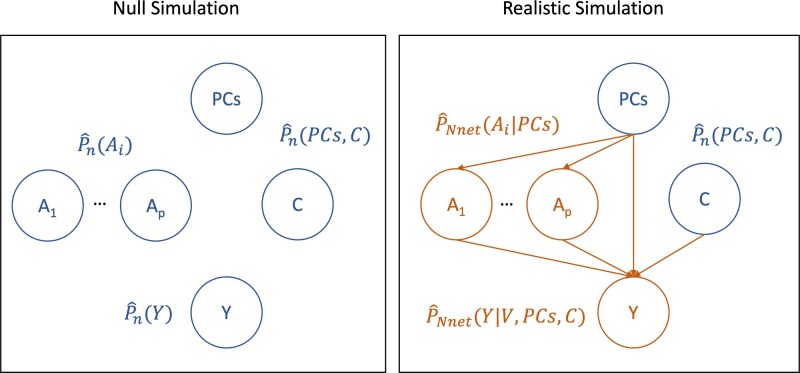
Generating processes of simulation studies. All distributions are empirical marginal distributions with the exception of the two learnt conditional densities in the Realistic Simulation (right). In both cases $ (PCs, C) $ are sampled jointly using the empirical marginal distribution. Left: In the null simulation, $ Y $ and $ A_{j} $ are sampled independently from their respective empirical marginal distributions. This results in the theoretical null hypothesis of no effect. Right: In the realistic simulation, each $ A_{j} $ is sampled from $ \hat{P}_{Nnet}(A_{j}|PCs) $, then $ Y $ is sampled from $ \hat{P}_{Nnet}(Y|A, PCs, C) $. $ \hat{P}_{Nnet} $ is described further in Section 2.2 of the Supplementary Materials. The various causal effects can then be approximated via Monte Carlo sampling. To connect with the previous section, the propensity score is given by $ g(A, PCs)=\prod_{i\,=\,1}^{p}\hat{P}_{Nnet}(A_{i}|PCs) $. The outcome regression model is defined as $ \bar{Q}(A, PCs, C)=\hat{P}_{Nnet}\left(Y|A, PCs, C\right) $ when $ Y $ is binary, and $ \bar{Q}(A, PCs, C)=\mathbb{E}_{\hat{P}_{Nnet}}\left[Y|A, PCs, C\right] $ when $ Y $ is continuous.

The first simulation, which we call the null simulation, examines conditions under which the proposed estimators appropriately control type I error rates when the null hypothesis of no effect is true ($ \Psi_{0}=0 $). The rationale behind this simulation is that most genetic variants are believed to have no or little effect (Rands et al. 2014; Boyle et al. 2017), and it is thus of particular importance that the type I error rate be controlled appropriately. Since we are interested in the effects of genetic variants on traits, the data generating process must satisfy $ Y\perp\!\!\perp A_{j} $ for all $ j\,=\,1, \ldots, p $ variants, and $ Y $ is a given trait. In practice, we use a stronger condition by making all these variables pairwise independent. We do this by independently sampling $ n $ times with replacement from each variable’s empirical marginal distribution. This generating process, presented in Fig. 1 (left), hence preserves many characteristics of the original dataset, while resulting in the true null hypothesis of no genetic effect on outcome.

The second simulation, termed the realistic simulation, exploits flexible conditional density estimators to fit an estimand-specific generating process illustrated in Fig. 1 (right). For example, the single variant effect of $ A_{1} $ on $ Y $ requires two density estimates, $ \hat{P}_{n}(A_{1}|PCs) $ and $ \hat{P}_{n}(Y|A_{1},PCs, C) $. Similarly, the interaction of $ (A_{1},A_{2}) $ on $ Y $ requires $ \hat{P}_{n}(A_{1}|PCs) $, $ \hat{P}(A_{2}|PCs) $ and $ \hat{P}_{n}(Y|A_{1},A_{2},PCs, C) $. To replicate the true data structure, the conditional density estimators must adequately reflect the complexity of the data. This has two key implications. First, the causal model should incorporate a rich set of causal variables to accurately generate downstream variables. To achieve this, we include in $ C $, genetic variants previously associated with $ Y $ by GeneATLAS (Canela-Xandri et al. 2018). Second, the density estimators must be sufficiently flexible and data-adaptive to model complex data-generating processes. For this, we use neural network-based estimators. Further details on variable and model selection are provided in the Supplementary Material. Given conditional density estimates, new data can be generated via ancestral sampling. We then verify that the conditions identified in the null simulation provide nominal coverage and estimate the power of discovery.

### 3.1. Considered estimands and estimators

We selected 25 estimands representative of common analyses in population genetics, focusing on single-variant and epistatic effects, quantified by the Average Treatment Effect (ATE) and Average Interaction Effect (AIE), respectively. Estimands were chosen to reflect realistic, non-null scenarios, though replicating true effect sizes was not the goal. All were supported by prior evidence of association. For single-variant effects, we selected five traits spanning a range of outcome types: leukocyte count, body mass index, sarcoidosis (rare, approximately 1,000 cases), multiple sclerosis (approximately 1,900 cases), and other digestive system diseases (ICD-10 K90–K93, approximately 25,000 cases). For each trait, two variants were manually selected from GeneATLAS (Canela-Xandri et al. 2018), requiring $ \mathrm{P-value} < 10^{-5} $. These variants were not in LD and had different minor allele frequencies. For epistatic effects, we selected variant combinations previously associated with specific traits: 2-point interactions for skin color (Morgan et al. 2018), a 4-point and 3-point interaction for Parkinson’s disease (Fernández-Santiago et al. 2019), a 2-point interaction for multiple sclerosis (Singhal et al. 2023), and a 2-point interaction for psoriasis (Singhal et al. 2023). These estimands span common and rare variants, as well as continuous, count, and binary traits. Full details are provided in Table 1. Together, they form a diverse benchmark to evaluate the performance of semi-parametric estimators in genetic analyses.

**Table 1.** Summary table of reproduced significant results for red hair color.

Variant 1	Variant 2	Effect-size	*P*-value
rs1805005 (GG $ \to $GT)	rs6059655 (GG $ \to $AG)	$ 1.8\times 10^{-2} $	$ 1.0\times 10^{-19} $
rs1805007 (CC $ \to $CT)	rs6088372 (CC $ \to $CT)	$ 3.0\times 10^{-2} $	$ 1.4\times 10^{-40} $
rs1805008 (CC $ \to $CT)	rs1129038 (TT $ \to $CT)	$ -1.9\times 10^{-2} $	$ 2.9\times 10^{-15} $
rs2228479 (GG $ \to $GA)	rs6059655 (GG $ \to $AG)	$ -1.6\times 10^{-2} $	$ 1.5\times 10^{-24} $
rs885479 (GG $ \to $GA)	rs6059655 (GG $ \to $AG)	$ -1.5\times 10^{-2} $	$ 1.6\times 10^{-16} $

We investigate the performance of the OSE and the wTMLE in both their canonical and cross-validated versions. For the estimation of nuisance functions, we consider two strategies: (i) a GLMNet-based approach (Friedman et al. 2010) incorporating all 2-point interaction terms involving genetic variants, (ii) an XGBoost-based approach (Chen and Guestrin 2016) where the $ \verb|(max_depth, lambda)| $ hyper-parameters are selected via cross-validation using a simple grid search. In all cases, the cross-validation scheme is a 3-fold cross-validation, stratified across the variants acting as treatment variables, and the outcome variables.

### 3.2. Results

To investigate the performance of semi-parametric methods on biobank-scale data, we perform the analysis across two different dataset sizes ($ 50\, 000,500\, 000 $). A simulation task is thus a triple: sample size, estimator, estimand. For each task, a grid of 500 bootstrap samples was run on the University of Edinburgh high-performance computing Eddie cluster.

#### 3.2.1. Null simulation

**Fig. 2.**
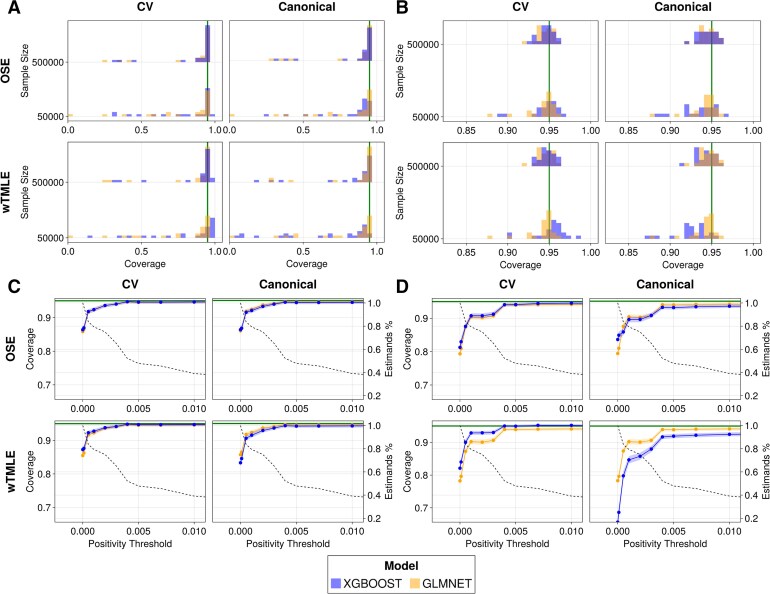
Null simulation coverage. Each plot is divided in 4 quadrants and presents coverage results. Rows correspond to estimators (OSE, wTMLE) and columns to resampling strategies (CV, Canonical). (A and B) Coverage distributions across estimates when (A) no positivity threshold is imposed and (B) a positivity threshold of $ \geq 0.01 $ is imposed. Each quadrant is sub-divided by sample size, $ 500\, 000 $ (top) and $ 50\, 000 $ (bottom). All estimators reach nominal coverage (green line) across most, but not all, estimands when no positivity threshold is imposed. Larger sample sizes yield better results and, when the positivity threshold of $ \geq 0.01 $ is imposed, nominal coverage for all four estimators is reached. The asymptotic regime has not been reached for all estimands at $ 50\, 000 $ samples. As the positivity threshold increases, the coverage reaches its nominal level as demonstrated in panels C and D. (C and D) Mean Coverage Across Positivity Thresholds [$ \mathbf{500\, 000} $ (C) and $ \mathbf{50\, 000} $ (D)]. A point corresponds to a positivity threshold (x-axis) and mean coverage across estimands (left y-axis). Only estimands’ components meeting the positivity threshold are included in the mean coverage computation. The black decreasing dotted line represents the percentage of total remaining estimands’ components after imposing the positivity threshold (right y-axis). For readability, the plots are limited to positivity thresholds $ < 0.01 $. Above this threshold, the nominal coverage was reached across all estimates.

Due to the absence of confounders in the null simulation of Fig. 1 (left), model misspecification is not expected to affect the estimation of the effect of genetic variants on outcome. All proposed estimators should thus provide asymptotic coverage at the nominal confidence level across all estimates (*e.g.*, 95%). Figure 2A shows that this is the case for most, but not all, estimands. For all four estimators (CV-)OSE and (CV-)wTMLE, there is at least one estimand for which the coverage is below 50%. This could be because the asymptotic regime has not been reached, possibly due to near-positivity violations for rare variants, noting that coverage is improved in the larger simulation. Exact control of positivity is challenging since it depends on the true, but unknown value of $ p(A\,=\,a|W\,=\,w) $ for all observed $ (a, w) $. In the null simulation however, $ p(A|W)=p(A) $ since $ A $ and $ W $ are sampled independently. We assess the impact of positivity by excluding estimands’ components that fail to meet a specified threshold in the original UK Biobank dataset, *e.g.*, $ p(A\,=\,a) < 0.01 $. Since estimands are multi-dimensional, representing various genotype changes, rather than discarding an entire estimand, we exclude only the genotype changes that do not meet the given threshold. For example, the average treatment effect of a variant is defined by two genotype transitions, conventionally denoted $ 0\to 1 $ and $ 1\to 2 $, which reflect increases in the number of effect alleles. If the effect allele is the minor allele, the genotype corresponding to two minor alleles will be the rarest in the population and may fall below the specified threshold. In such cases, the $ 1\to 2 $ transition is discarded and the average treatment effect is computed for a single genotype transition, $ 0\to 1 $. This approach is more conservative than the data-adaptive selection criterion for the propensity score truncation level proposed in (Gruber et al. 2022).

In Fig. 2B, coverage estimates are shown for those estimands whose components have been pruned to satisfy the threshold $ p(A\,=\,a)\geq 0.01 $, and coverage is seen to be near nominal. Furthermore, by varying the positivity threshold value of 0.01, we analyze the estimators’ sensitivity to practical positivity violations. Figure 2C and D present coverage results across various positivity thresholds for sample sizes $ 500\, 000 $ (C) and $ 50\, 000 $ (D) respectively. As anticipated, convergence to nominal coverage is faster for the larger sample size. However, for both sample sizes, on average, nominal coverage is achieved for all genotypes with frequencies as low as 0.005. This holds true across all estimators, except for the canonical wTMLE using XGBoost when the sample size is $ 50\, 000 $. The likely cause of this deviation is overfitting, as the cross-validated estimator restores coverage to the desired level. The percentage of remaining estimands drops rapidly with increasing positivity thresholds. For the conservative 0.01 threshold, approximately 40% of estimands’ components remain.

While of primary importance to researchers, coverage provides an incomplete view of an estimator’s performance. We additionally analyze the bias-variance decomposition across all estimation tasks. Squared bias, variance and mean-squared error are estimated via bootstrap resampling as per Section 2.6 and provided in Table S6. Here, the variance is not estimated using the influence function but as the sample variance of estimates on bootstrapped data. However, these are comparable provided a sufficient positivity constraint is applied (see Fig. S4). For direct comparison, the variance of the influence function is also provided in Table S6. For the null simulation, these are almost always similar (less than 15% relative difference) for both sample sizes when using GLMNet, and for the larger sample size $ 500\, 000 $ when using XGBoost. As expected, the bias and variance of estimators decrease with sample size for almost all estimands. The only 4 exceptions (out of 100), occur for the rarest trait in our dataset (sarcoidosis).

Interestingly, when using XGBoost, the bias of cross-validated estimators is always larger than that of their canonical counterpart. This unexpected result seems to indicate that the asymptotic regime is not reached, possibly due to the low number of folds used throughout ($ k\,=\,3 $). Effectively, each nuisance function is fitted on $ 2/3 $ of the data, increasing the risk of overfitting on each partition. This limitation could be overcome by increasing the number of folds. However, the increased computational complexity is challenging in population studies of this scale. Inspecting results from the same analysis when using GLMNet, a model less prone to overfitting, for nuisance function estimation, shows that bias estimates are more varied between the canonical and cross-validated estimators. This observation supports the potential explanation for higher bias in cross-validated estimators when using a more flexible algorithm such as XGBoost for nuisance function estimation.

Together, these results demonstrate that in the absence of genetic effects, the false discovery rate can be appropriately controlled provided the minor allele frequency is at least 0.01. The cross-validated estimators implemented in TarGene can be used to trade bias and variance for improved coverage.

#### 3.2.2. Realistic simulation

We now turn to the results of the realistic simulation, where genetic variants influence traits, potentially in complex ways, as modeled by the Sieve Neural Network Estimator (SNNE) Algorithm 1. The empirical performance of SNNE across different densities is shown in Fig. S1, demonstrating that this approach offers a flexible, data-adaptive solution for modeling complex data-generating processes while mitigating the risk of overfitting.

**Fig. 3.**
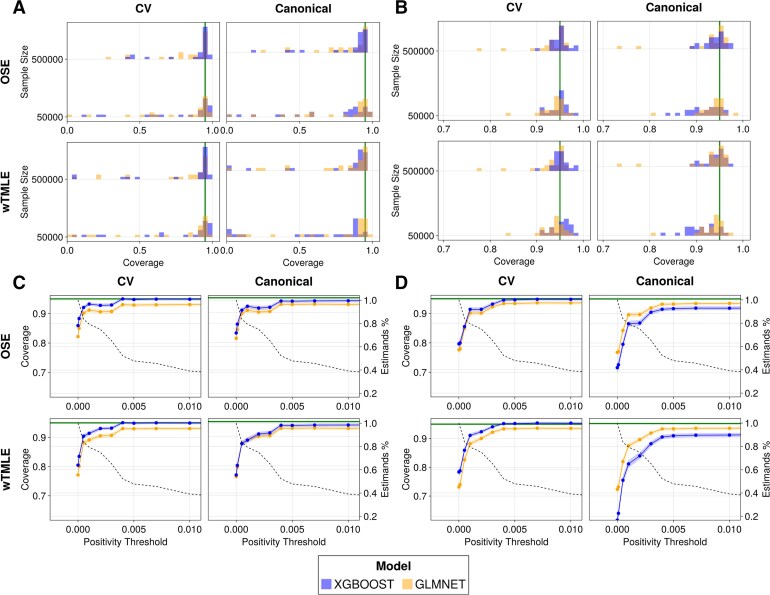
Realistic simulation coverage. The panel is organized exactly as Fig. 2. (A and B) Coverage distributions across estimates when (A) no positivity threshold is imposed and, (B) a positivity threshold of $ \geq 0.01 $ is imposed. Similarly to Fig. 2, larger sample sizes yield better results, more so for the smaller sample size of $ 50\, 000 $ indicating that the asymptotic regime has not been reached for all estimands. As the positivity threshold increases, the coverage reaches its nominal level as demonstrated in panels B and C with the exception of the canonical OSE and wTMLE at sample size $ 50\, 000 $. (C and D) Mean Coverage Across Positivity Thresholds [$ \mathbf{500\, 000} $ (C) and $ \mathbf{50\, 000} $ (D)]. In large sample settings, XGBoost-based estimators perform better than their GLMNet counterpart. For smaller sample-sizes, cross-validation is preferred for these models as they are more prone to overfitting.

**Fig. 4.**
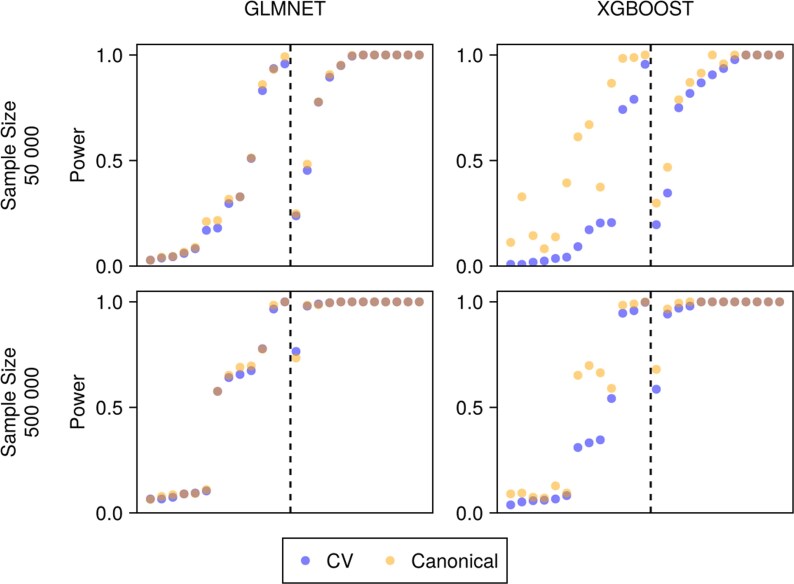
Power analysis of weighted TMLE. The plot is organized in four quadrants. Rows indicate sample sizes with $ n\,=\,50\, 000 $ (top) and $ n\,=\,500\, 000 $ (bottom), columns indicate the model used to fit nuisance functions with GLMNet (left) and XGBoost (right), and color indicates the resampling scheme, ie cross-validated (blue) and canonical (orange). Each dot corresponds to a single estimand and the dashed lines separate AIEs (left) and ATEs (right).

The issue of positivity, discussed in the previous section, persists in the realistic simulation, as shown in Fig. 3A, and is similarly resolved when imposing a positivity threshold $ p(A\,=\,a)\geq 0.01 $ as shown in Fig. 3B. This suggests that the dependence on $ PCs $ is mild and does not need to be taken into account in the positivity criterion, hence also justifying the minor allele frequency threshold traditionally used in GWAS. Figure 3C and D indicate that for the same positivity threshold of 0.005, nominal coverage is achieved when cross-validated estimators are combined with XGBoost. This is likely due to two factors: (i) XGBoost effectively captures the complexity of the true data-generating process, and (ii) while XGBoost tends to overfit, cross-validation compensates for this tendency. In large datasets, XGBoost consistently provides better coverage than GLMNet, regardless of the resampling strategy. In settings such as UKB, the canonical version of XGBoost-based estimators offers an interesting trade-off between computational efficiency and coverage, with only a marginal $ \approx 1\% $ drop. However, in smaller datasets, XGBoost underperforms when cross-validation is not used, likely due to overfitting. The analysis of bias, variance and mean squared error, similar to that of the null simulation, mostly leads to the same conclusion; see Table S6 for full results. The improved coverage of CV estimators is largely gained at the expense of bias and variance when models prone to overfitting are used. The sample variance and variance of the influence function are more likely to differ (ie more than 15% relative difference) at the smaller sample size of $ 50\, 000 $ when using XGBoost; the difference is comparable between sample sizes when using GLMNet. This suggests model misspecification plays a larger role in the realistic simulation than in the null simulation where treatment is randomly assigned, as expected.

In tandem with nominal coverage, the power of wTMLE across estimands, sample sizes, resampling schemes and models used to estimate nuisance functions is presented in Fig. 4. A similar plot is presented for the OSE in Fig. S2. As expected, increased sample sizes result in higher power for both models. Interestingly, cross-validation affects the power of the XGBoost model but not the GLMNet model. This suggests that in the absence of overfitting there is no or little loss of power from cross-validated estimators. Furthermore, this analysis shows that the power to detect ATEs (right of the dashed lines) is larger than the power to detect AIEs (left of the dashed lines). This is expected since the variance, and hence power, of an estimator depends on the complexity of the estimand.

In the simulation presented here, the interacting genetic variants were chosen to be independent given observed confounders. However, in population genetics, correcting for population stratification is not necessarily sufficient to account for confounding due to LD. To demonstrate that our estimators maintain performance when interacting variables are in LD, we present an additional simulation in Fig. S3 in which variants $ A_{1} $ and $ A_{2} $ are confounded by population stratification and LD, and both cause $ Y $.

## 4. APPLICATION TO STATISTICAL GENETICS AND LARGE BIOBANKS

We performed a series of analyses using (i) the UK Biobank (UKB) cohort (Bycroft et al. 2018), and (ii) the *All of Us* (AoU) cohort (Bick et al. 2024) to demonstrate the performance of semi-parametric estimators in the context of real-world genotype-phenotype inference. For comparison with previous studies such as the GeneATLAS (Canela-Xandri et al. 2018), all analyses presented here were performed on the subpopulation of individuals with a self-reported white ethnic background. Specifically, in UKB we selected individuals with data field 21000 subcoding in the category 1 (“White”), namely 1001 (“British”), 1002 (“Irish”), and 1003 (“Any other white background”). In the AoU cohort, we included only individuals from non-Hispanic or Latino descent and inferred European genetic ancestry.

First, we contrast our approach with the beta-coefficient of the commonly used LMM by performing a phenome-wide association study on UKB for a well studied variant in the *FTO* gene region: rs1421085. We compare the estimated effect of this SNP on BMI in the UKB with the corresponding estimate in AoU. Second, we reveal gene-by-environment interactions between rs1421085 and two deprivation indices. Third, we replicate pairwise genetic interactions previously reported for hair color by Morgan et al. (2018) and report additional evidence of interactions for both skin and hair color. And fourth, we show how TarGene can investigate higher-order interactions using multiple loci related to vitamin D receptor (VDR) function.

### 4.1. Setting

For all analyses we used the three canonical estimators, ie TMLE, wTMLE and OSE. The nuisance functions $ Q $ and $ g $ were estimated using the practical recommendations for Super Learning (Phillips et al. 2023). In brief, we use (i) $ k $-fold cross-validation or stratified $ k $-fold cross-validation based on the outcome type (continuous or binary, respectively), here $ 3\leq k\leq 20 $, selected adaptively based on the rarest class of each outcome, and (ii) included the constant fit, a regularized logistic/linear regression (ridge, $ \lambda\,=\,1 $), a gradient-boosted tree ($ \verb|n_round|=100 $, default parameters otherwise), and HAL with hyper-parameters max_degree  $ =1 $, smoothness_orders  $ =1 $, lambda  $ =30 $ (Benkeser and van der Laan 2016), as base learners. However, we note that for the optimal performance of HAL in more bespoke analyses, the parameter $ \lambda $, bounding the total variation norm, should be left unspecified so that it is chosen by the algorithm’s internal cross-validation. For confounding adjustment, we used the first 6 PCs computed from the genotypes. We also added age at assessment and genetic sex as explanatory variables for the outcome model $ \hat{Q} $. To investigate the impact of SP variance correction, variance estimates were corrected using 100 genetic similarity thresholds $ \tau $. Full run configurations can be found .

### 4.2. *FTO* PheWAS

We conducted a phenome-wide association study (PheWAS) using UKB data for 768 traits. We compared our results with those from the LMM analysis of Canela-Xandri et al. (2018). For a comparison across populations, we contrast TarGene’s UKB results on BMI with the corresponding results on BMI when running TarGene on the AoU cohort. We chose a well-studied variant, rs1421085, located in the first intron of the *FTO* gene. For this variant, the T-to-C nucleotide substitution has been predicted to disrupt the repression of *IRX3* and/or *IRX5*, thereby leading to a developmental shift from adipocyte browning to whitening programs and loss of mitochondrial thermogenesis (Claussnitzer et al. 2015). This variant has also been associated with several related traits, such as BMI and obesity (Frayling et al. 2007). Since rs1421085 has two different alleles, the three possible genotype changes we estimate for each trait are (i) TT $ \to $ TC, (ii) TC $ \to $ CC, and (iii) TT $ \to $ CC. If C is the effect allele, these transitions are equivalent to the conventional $ 0\to 1 $, $ 1\to 2 $ and $ 0\to 2 $ notation.

In general, the effect of the first C substitution (TT $ \to $ TC) on trait $ Y $ may differ from the effect of the second substitution (TC $ \to $ CC). When the assumption of equal effects fails, standard linear models, estimating a single additive effect, will capture only a compromise between the two. Although additive-dominance models (Palmer et al. 2023) allow for such distinctions, they are rarely used in large-scale association studies. In contrast, our proposed framework treats each allelic change as a first-class citizen, enabling direct and separate estimation of their effects. In what follows, we first compare GeneATLAS’ effect sizes to our TT $ \to $ TC estimate. We then explicitly test the existence of Allelic Effect Difference ($ \psi_{\Delta} $) using the estimator of (21).

#### 4.2.1. Comparing effect sizes between methods

**Fig. 5.**
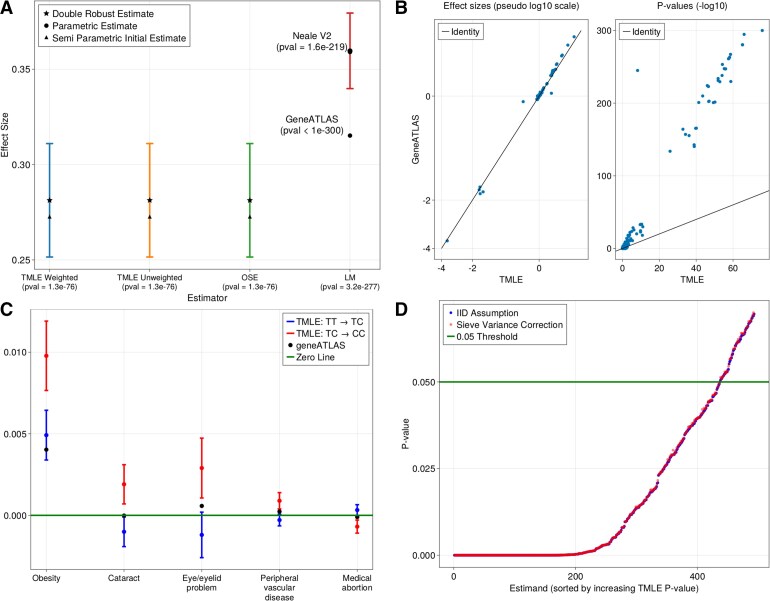
TarGene results and comparison with LMM estimates for FTO SNP rs1421085 in UKB. (A) Inference results. Comparison of effect size estimates of rs1421085 on body mass index (BMI; UKB data field 23104). The three double robust estimators share the same initial fit but apply different targeting strategies: weighted TMLE (blue), unweighted TMLE (orange), and OSE (green). Neale V2 and GeneATLAS use a linear model and LMM, respectively, but do not report standard deviations. We refit the Neale V2 linear model to obtain a confidence interval (red). The central value of GeneATLAS does not lie within the 95% confidence interval of Neale V2. In contrast, the three double robust estimates are concordant and exhibit a statistically significant lower effect size than the linear model based inference. See Fig. S7C for a comparison between the initial estimates of effect sizes, relying on Super Learning, against the final effect sizes after the TMLE step. (B) Comparison with GeneATLAS. Comparison of effect sizes (left) and *P*-values (right) reported by TarGene (TMLE) and GeneATLAS (LMM). Effect sizes are concordant overall on this study but the *P*-values reported by TarGene are more conservative. Because TarGene’s *P*-values are more robust to model misspecification, GeneATLAS’ results likely contain an inflated set of false discoveries. (C) Non-linear effects. A selection of traits for which rs1421085 TT $ \to $ TC and TC $ \to $ CC effect estimates are significantly different; Table S2 contains the complete list. Effect sizes are reported with associated 95% confidence intervals together with estimates from GeneATLAS’ LMM fits (black points) ( Canela-Xandri et al. 2018). The latter almost always fall in-between TarGene’s TT $ \to $ TC and TC $ \to $ CC estimates, indicative of an averaging effect. (D) Sieve variance correction. *P*-values obtained from two variance estimation methods for rs1421085. In red, the UKB participants are assumed to be iid, while in blue, a sieve correction method is applied to account for possible population dependence structure. Each *P*-value corresponds to a specific parameter of interest for which the initial iid estimate was under the $ P\,=\,0.07 $ threshold. See Fig. S7A and B for the unfiltered plot and an example of a Sieve Plateau curve, and Panel D for the histogram of the GRM.

We discuss the difference between our reported TT $ \to $ TC effect on BMI, and the effects present in GWAS catalogues (Buniello et al. 2019). PCs on the UKB white population were computed and are shown in Fig. S5. The results in Fig. 5A show that all three double-robust estimators are concordant and report statistically lower effect sizes than the two linear (mixed) models which, in contrast, are discordant with one another. Apart from BMI, semi-parametric point estimates are mostly aligned with those produced by LMMs (Fig. 5B, left). This is expected since in the absence of confounding, effect estimates with linear models are robust to model misspecification, offering reliable estimates even when the true relationship between variables is not linear. For rs1421085, the absence of confounding by PC‘s is shown in Fig. S6. Therefore, for this variant, the main source of differences between semi-parametric and linear estimates will likely be due to allelic effect differences.

Furthermore, Fig. 5B shows that the distribution of *P*-values is shifted toward less significant values as compared to GeneATLAS estimates. After FDR correction across the 768 traits, TarGene finds fewer significant results at the 0.05 level (63 traits) than GeneATLAS (159 traits). This phenomenon highlights the risk of an increased false discovery rate due to overly restrictive parametric assumptions. A summary table of all significant estimation results after multiple testing adjustment is provided in Table S1.

#### 4.2.2. Allelic effect differences

We find 39 traits for which rs1421085 displays a significant Allelic Effect Difference, 35 of which are highly correlated with BMI. For instance, we find that the departure from TT to TC is associated with an increased weight (UKB field 21002) of 0.78 kg (95% CI: $ 0.69-0.86 $ kg). In comparison, the departure from TC to CC is associated with a significantly larger increase of 1.33 kg (95% CI: $ 1.20-1.45 $ kg). This is concordant with previous research showing that variants in the FTO locus have recessive effects on risk of obesity (Wood et al. 2016). For illustration, a subset of significant non-linear traits is presented in Fig. 5C; Table S2 contains the complete list. As might be expected, most estimates reported by GeneATLAS, based on a LMM approach, fall in-between estimates from our two scenarios, representative of an averaging effect. Notably, some traits display opposite effect sizes (before multiple testing correction) for the allelic changes TT $ \to $ TC and TC $ \to $ CC. Thus TarGene can capture variant-trait pairs displaying heterozygote advantage (Hedrick 2012). Such patterns cannot be detected by a linear model that assumes equal allelic effect sizes.

#### 4.2.3. Sieve plateau variance estimation

To account for the dependence among individuals when estimating the variance of our estimators, we use the SP variance estimation of (Davies and van der Laan 2014). The SP method is computationally intensive, requiring (i) computation of the GRM, an $ N\times N $ matrix (for UKB, $ N\approx 450\, 000 $), and (ii) for each estimand and each threshold $ \tau $, matrix multiplications involving GRM components and influence curves. However, since SP only increases variance estimates, it is sufficient to consider estimates that are significant at a given threshold (*e.g.*, $ P\,\lt\,0.05 $) before SP variance correction. In Fig. 5D, we report the *P*-values resulting from the iid (red) and the SP (blue) variance estimates for all effect sizes obtained for rs1421085 (with initial $ P\,\lt\,0.07 $ for improved readability), which show little difference. An example SP variance curve for the effect estimate of rs1421085 on BMI shows an increased variance of $ \approx 1.4\% $ (Fig. S7). The covariance term of influence functions of related individuals are thus likely negligible as compared to the individual variance terms in (24). The impact of the SP variance method in a more diverse or related population is an interesting research direction.

#### 4.2.4. Comparing effect sizes between cohorts

Next, we investigated whether our UKB results replicate in another large biobank, noting potential differences in allelic frequencies and environmental factors. To explore this, we leveraged the *All of Us* (AoU) cohort (Bick et al. 2024), a United States-based cohort. This study was performed on AoU’s cloud-based platform called the Researcher Workbench, which is compatible with the TarGene software.

We constructed a cohort with criteria that matched our UKB study, requiring that each participant had complete data available for BMI, age at BMI measurement, sex at birth, and genetics. We included only individuals of inferred European genetic ancestry and of non-Hispanic or Latino origin to mirror inclusion criteria for our UKB study. Principal components were computed across all remaining participants ($ n\,=\,122,752 $) and genotyped variants, excluding any variants in LD with our variant-of-interest, rs1421085. In this cohort, 6 PCs were sufficient to account for confounding due to population stratification, see Fig. S8.

In the AoU cohort, we found that rs1421085 also had a significant effect on BMI for each allelic change, consistent with results in UKB. While TT to TC was associated with an increase of 0.59 kg/m$ {}^{2} $ (95% CI: $ 0.42-0.77 $, $ P\,\lt\,1\times 10^{-10} $), TC to CC was associated with an increase of 0.88 kg/m$ {}^{2} $ (95% CI: $ 0.47-1.28 $, $ P\,\lt\,1\times 10^{-4} $). Estimates in the AoU cohort were larger than those in UKB, but trends were consistent between cohorts. The effect of the allele change TC $ \to $ CC on BMI was also shifted higher than TT $ \to $ TC, however, estimates in the AoU cohort harbored higher levels of uncertainty, and so no significant non-linear effect was found between individual allele changes in AoU (*P*-value $ =0.27 $), see Fig. S9. The increased uncertainty may be due to the smaller sample size of our AoU cohort ($ n\,=\,122,752 $) relative to the UKB cohort ($ n\,=\,459,207) $, a decrease in sample size (to $ \approx 27\% $ of UKB) and minor allele frequency for the C allele ($ \approx 10\% $ lower) in AoU, resulting in substantially reduced sample sizes for the CC genotype, see Fig. S9. Alternatively, this effect may also be due to increased variation of BMI in the AoU cohort due to environmental effects.

### 4.3. Gene-by-environment interaction

In the previous section, our TarGene PheWAS on UKB confirmed that rs1421085 is significantly associated with BMI and various BMI-related traits. This association between rs1421085 and BMI was also found to be replicated in the *All of Us* cohort. Since BMI has also been associated with area-based deprivation (Twaits and Alwan 2020), it is natural to investigate the potential interactions between rs1421085 and deprivation. There are currently two main measures of deprivation in the UK: the Townsend Deprivation Index (TDI) and the Index of Multiple Deprivation (IMD) used by (Twaits and Alwan 2020). We used these indices in two separate phenome-wide interaction studies (PheWIS), one between rs1421085 and TDI and one between rs1421085 and IMD. Since deprivation indices are continuous quantities, we discretized them using quintiles and compared the most extreme quintiles. For rs1421085, we compare the three genotype groups TT, TC, CC.

We found 21 significant BMI related traits ($ {\rm FDR} < 0.05 $) captured by both TDI and IMD (Table S3). For instance, whilst we have seen that an increase in the number of C alleles in an individual is associated with an increase in body weight and that most deprived individuals are more likely to be overweight, the interaction of these factors is super additive: an increase of 1.07 kg (*P*-value: $ 1.09\times 10^{6} $, adjusted *P*-value: $ 1.4\times 10^{-3} $) for TDI, and an increase of 0.91 kg (*P*-value: $ 3.89\times 10^{-5} $, adjusted *P*-value: $ 8.0\times 10^{-3} $) for IMD.

### 4.4. Epistatic interaction

Detection of epistasis in complex traits is challenging (Wei et al. 2014; Mackay and Anholt 2024). For example, epistatic interactions are expected to be much smaller than main effect sizes, which can already be small for polygenic traits. In this section, we explore the potential for semi-parametric estimators to reveal such interactions. For that purpose, we rely on a study investigating hair color, in which nine pairs of variants were reported to be statistically interacting with red-hair using a logistic regression model and a likelihood ratio test (Morgan et al. 2018). We note, however, that the likelihood ratio test statistic measures interactions on a multiplicative scale while we investigate interactions on an additive scale which is often of more direct public health relevance (VanderWeele and Knol 2014). In particular, the existence of interactions on one scale does not imply the existence of interactions on the other scale.

We found that five of the nine reported epistatic results (Table 1) are also revealed by semi-parametric estimation methods. Two were not reproduced and two were not computed because they did not pass the marginal positivity threshold (0.01). We further find 27 significant epistatic signals for traits corresponding to either skin or hair color (Table S4). This is expected because hair and skin colour are known to co-vary (Sulem et al. 2007).

### 4.5. Exploration of new epistatic loci

Finally, we show how TarGene can investigate any complex biological mechanisms by estimating higher-order interactions. As an illustration, we focus on VDR, a nuclear hormone receptor that binds 25-hydroxyvitamin D (25OHD), the active form of vitamin D, and the retinoid-X receptor (RXRA). This complex can then enter the nucleus and regulate gene transcription programmes. Because this mechanism depends on three interacting molecules, it provides a natural field for investigating epistasis. Three genetic variants were identified that have previously been associated with differential expression of each molecule: A to C (rs7971418) is associated with increased levels of VDR mRNA; G to T (rs1045570) is associated with increased levels of RXRA mRNA eQTLGen,; and, C to T (rs3755967) has been associated with decreased 25-hydroxyvitamin D (jiangGenomewideAssociationStudy2018). Although no interaction between these variants was detected after FDR adjustment (FDR $ < 0.05 $, Table S5); 47, 42 and 39 pairwise interactions, and 21 3-point interactions were significant in single tests, hence demonstrating the potential of TarGene.

## 5. SOFTWARE AND PIPELINE

We release two open-source packages and one software for scalable semi-parametric estimation of causal effects.

### 5.1. General purpose package and CLI

TMLE.jl is a Julia package for the semi-parametric estimation of causal effects from tabular datasets. The package currently supports one-step and targeted minimum loss-based estimation of the counterfactual mean, the average treatment effect (ATE), the average interaction effect (AIE), and any differentiable function thereof. The package also supports multi-dimensional and categorical treatment variables. The associated TMLECLI.jl provides an executable command-line interface.

### 5.2. Population genetics nextflow pipeline

We also provide the domain specific TarGene software, a scalable Nextflow pipeline for semi-parametric estimation of genetic effects from population cohorts. TarGene can be run seamlessly on two major databases: (i) the UK Biobank (downloaded data) (Bycroft et al. 2018), and (ii) the cloud-based *All of Us* Researcher Workbench (Bick et al. 2024). Inbuilt support for future use cases, such as the upcoming cohort Our Future Health, will be added when available. TarGene supports multiple workflows and study designs: Genome-wide association study (GWAS), Phenome-wide association study (PheWAS), and custom study design of (i) single or joint variant effects on outcome, (ii) gene-by-gene ($ G\times G $) effects up to any order, and (iii) gene-by-environment ($ G\times E $) effects up to any order.

We show below that computationally intensive PheWAS and GWAS studies are possible on modern computing resources. In our case, all runs were performed on the Edinburgh high-performance Eddie cluster. While TarGene comprises standard pre-processing procedures, we focus on the two unique processes: (i) the semi-parametric estimation process and (ii), the sieve variance plateau estimation process.

#### 5.2.1. Semi-parametric estimation process

In this section, we investigate the run time of the TMLE process for the two most common genetic studies: GWAS and PheWAS. In both cases, we are thus computing the Average Treatment Effect for each individual variant on trait by comparing the major/minor to the major/major genotype. Covariates were set to include the first 6 principal components, age and sex. The benchmark is performed on a single core compute node.

We investigate the following four nuisance parameters estimation strategies (applied to both the outcome regression $ \bar{Q} $ and the propensity score $ g $) from the most basic to the most comprehensive:

• GLM: Standard generalized linear model
• GLMNet: GLM with regularization hyperparameter tuning over 3-folds cross-validation.
• XGBoost: The gradient boosting trees method with hyper-parameter tuning over 10 different settings in a 3-folds cross-validation scheme.
• SL: Super Learning including both XGBoost and GLMNet combined with an outer 3-folds cross-validation.

We first focus on the PheWAS setting for which run time estimates are provided in Table 2. In a PheWAS the estimation of the propensity score, $ g $, only needs to be performed once and can be re-used across all traits. The computational complexity is thus driven by the estimation of each regression, $ \bar{Q} $, and associated targeting steps. The same remark holds for the targeting steps corresponding to the various genetic changes. Computing the effects of the additional major/minor $ \to $ minor/minor and major/major $ \to $ minor/minor only costs two additional targeting steps while re-using the current $ \bar{Q}_{n} $. In all cases, running a PheWAS using TarGene is feasible even without access to a high-performance computing platform.

**Table 2.** PheWAS run times for various nuisance parameters estimation strategies.

Learning algorithm	Time (h)
GLM	2.2
GLMNet	4.5
XGBoost	8.8
SL	30

We now turn to the GWAS setting for which run time estimates are provided in Table 3. In this case, $ \bar{Q} $ and $ g $ need to be estimated for each variant. In order to obtain a run time estimate for a GWAS across genotyped loci ($ \approx 600\, 000 $ variants), we run the TMLE process over 100 variants and report the scaled mean run time. While it is not currently possible to run a GWAS on a personal laptop, we find that access to a modern computing platform makes this kind of study feasible using TarGene.

**Table 3.** GWAS run times. The unit time corresponds to a single variant/trait pair. The projected GWAS time assumes $ 600\, 000 $ variants and 200 folds parallelization.

Learning algorithm	Mean time (seconds/variant)	Projected GWAS time (h)
GLM	13	10
GLMNet	57	48
XGBoost	95	72
SL	451	375

#### 5.2.2. Sieve variance Plateau process

We present benchmarks for Sieve Variance Plateau estimation on a 20-core compute node in Table 4. For each parameter, the procedure computes a given number of variance estimates, here either 10 or 100 $ \tau $-values. Table 4 shows computational time increases sub-linearly with both the number of estimates and the number of parameters. This is mainly because, for a small number of parameters, the computational time is driven by reading the GRM from disk hence under-using the multi-threading power of the node. As soon as the number of parameters becomes large, *e.g.*, for a GWAS or PheWAS, all cores can be utilized simultaneously to maximum efficiency. Due to the computational cost of the SVP correction, we recommend applying it only to estimates that fall just below the desired significance threshold.

**Table 4.** Sieve Plateau variance estimation benchmarks.

Number of estimates per curve	Number of parameters	Time (s)	Time (h)
100	419	71836	19.9
10	419	11926	3.3
100	1	30966	8.6
10	1	6779	1.8

## 6. DISCUSSION

We have introduced Targeted Genomic Estimation (TarGene), a method based on targeted semi-parametric estimation theory (Pfanzagl and Wefelmeyer 1985; van der Laan and Rose 2011) for the estimation of genetic effects of single variants and interactions. TarGene offers a number of distinct advantages over commonly used parametric approaches as it avoids model-misspecification bias, produces asymptotically normal and efficient estimates, and is doubly-robust. Due to the flexibility of its SL libraries and the TMLE step, it can be readily applied to more ancestry heterogeneous biobanks such as *All of Us* (Bick et al. 2024) or the *Million Veterans Program* (Gaziano et al. 2016), as well as more strongly inter-related cohorts such as island communities. We have demonstrated in extensive realistic and data-adaptive simulations that TarGene achieves nominal coverage and control of type I error, provided the minor allele frequency is bounded from below by 0.01.

Whilst TarGene’s run time is slower than some other GWAS estimation methods, the strength of TarGene lies in bespoke analyses of effect sizes and interactions among targeted variants of interest, providing mathematically guaranteed coverage of the ground truth. For researchers aiming to apply TarGene in large-scale settings, we recommend a two-stage analysis strategy. In the first stage, computationally efficient learners—such as linear models (e.g., GLMnet)—can be used to estimate nuisance functions. The lightest configuration of TarGene completes a genome-wide scan of approximately 600,000 variants in about 10 h on a high-performance computing (HPC) cluster (see Table 3). In the second stage, significant associations identified in the initial scan can be re-evaluated using a more flexible TarGene configuration that leverages a stacked Super Learner library. Taking advantage of the approach described by Tuglus and van der Laan (2009) nevertheless allows the researcher to control the final FDR of this two-stage procedure. We also recommend applying the SP variance correction to estimates with *P*-values near the FDR threshold to ensure their robustness.

The estimators developed and implemented in this work take into account population stratification, via genetic ancestry principal component analysis (PCA), as a confounder of the variant-to-outcome relationship, as well as covariates, such as age and sex, which can influence outcome. However, in general, this does not imply that the estimated genetic effect sizes reflect causal mechanisms. This is because of the co-inheritance of dependent variants, known as linkage disequilibrium (LD). While TarGene estimators address any statistical gap due to model misspecification, the causal gap currently remains. Attempts have been made to address the causal gap in population genetics analyses through fine-mapping (Benner et al. 2016; Wang et al. 2020; Zou et al. 2022; Zeng, 2024). These methods, most of which developed in the Bayesian framework, also rely on parametric assumptions and may not close the statistical gap. Methods based on the knockoff filter of Foygel and Candès (2015), such as KnockOffGWAS (Sesia et al. 2021), represent a promising avenue for the detection of causal variants but do not quantify effect sizes or epistatic interactions (Guan and Levy 2025). This quantification is essential for explaining how variants, via biological mechanisms and regulatory functions, modify a trait or disease risk. A unified method that closes both causal and statistical gaps in genomic medicine has yet to be developed.

In future work, we will investigate various collaborative TML estimators (CTMLE) (van der Laan and Gruber 2010; Ju et al. 2019) and adaptive TML estimators (ATMLE) (van der Laan et al. 2025) to reduce the causal gap due to LD. CTMLE has been applied successfully in situations with many potential confounders, see *e.g.*, Schnitzer et al. (2016); Pirracchio et al. (2018). Additionally, we plan to explore non-linearities (Palmer et al. 2023) in variant allelic copies on trait using TarGene. We will also investigate the contribution of epistatic interactions of specific variants on various polygenic traits for a variety of biological mechanisms.

## Supplementary Material

kxaf030_Supplementary_Data

## Data Availability

This study used data from the *All of Us* Research Program’s Controlled Tier Dataset v7, available to authorized users on the Researcher Workbench.
