# Supplementary material for: Semiparametric efficient estimation of small genetic effects in large-scale population cohorts
Source: Biostatistics. 2025 Sep 30;26(1):kxaf030. doi: 10.1093/biostatistics/kxaf030 (PMC12479317; doi:10.1093/biostatistics/kxaf030)
Supplement: kxaf030_Supplementary_Data [file kxaf030_supplementary_data.zip › biosts-25031-File002.pdf]

Journal Title Here Vol. , No. , pp. 1–21
doi: DOI HERE
Advance Access Publication Date: Day Month Year
Paper

Supplementary Materials for "Semiparametric efficient estimation of small genetic effects in large-scale population cohorts"

Olivier Labayle,[1,2] Breeshey Roskams-Hieter,[3,4] Joshua Slaughter,[1] Kelsey Tetley-Campbell,[4] Mark J. van der Laan,[5] Chris P. Ponting,[4] Sjoerd V. Beentjes[4,5,6,†,*] and Ava Khamseh[1,4,5,†]

[1]School of Informatics, University of Edinburgh, 10 Crichton Street, EH8 9AB, Edinburgh, United Kingdom, [2]Institute for Regeneration and Repair, University of Edinburgh, 4–5 Little France Drive, EH16 4UU, Edinburgh, United Kingdom, [3]Health Data Research UK, 215 Euston Road, NW1 2BE, London, United Kingdom, [4]MRC Human Genetics Unit, Institute of Genetics and Cancer, University of Edinburgh, Crewe Road South, EH4 2XU, Edinburgh, United Kingdom, [5]School of Public Health, University of California, Berkeley, 2121 Berkeley Way, 94720, California, United States of America and [6]School of Mathematics and Maxwell Institute for Mathematical Sciences, University of Edinburgh, Peter Guthrie Tait Road, EH9 3FD, Edinburgh, United Kingdom

*Corresponding author. Email: Sjoerd.Beentjes@ed.ac.uk
†These authors contributed equally to this work, alphabetical order.

FOR PUBLISHER ONLY Submitted Date Month Year; Revised Date Month Year; revised version accepted Date Month Year

ABSTRACT

These supplementary materials contain the derivation of the efficient influence function, exact remainder, and corresponding One-Step and Targeted Minimum Loss-based Estimators for our k-point interaction parameter, as well as further simulations, details and figures.

PROPERTIES OF THE K-POINT INTERACTION PARAMETER

In this section, we first establish the statistical properties of the k-point interaction parameter formally, including the double robustness of its influence function. We then give a precise description and implementation of the One-Step Estimator (OSE), the (weighted) Targeted Minimum Loss-based Estimator ((w)TMLE), and their cross-validated (CV) counterparts, including their respective loss functions. We formally verify that the weighted fluctuation underlying the weighted TML Estimator solves the efficient influence function. Finally, we describe the CV variance estimators and the sampling splitting schemes.

Inferential problem

Recall the observed data unit $O = (W, A_1, A_2, \ldots, A_m, Y) \sim P_0$, where W is a vector of pre-treatment covariates, the $A_i \in \{0, 1, \ldots t_i\}$ with $i = 1, \ldots, m$ are categorical treatment variables, Y is an outcome of interest, and $P_0 \in \mathcal{M}$ is the true data-generating probability distribution. While our derivations carry through under arbitrary restrictions on the distributions of the A_i given W, we work with the non-parametric model $\mathcal{M} = \mathcal{M}_0$ throughout so as to not make potentially unrealistic assumptions on $P_0 \in \mathcal{M}$.

© The Author 2022. Published by Oxford University Press. All rights reserved. For permissions, please e-mail: journals.permissions@oup.com

Our statistical estimand of interest is the k-point interaction among a subset of the treatment variables $A_1, \ldots, A_m$ in their effect on outcome Y whilst correcting for covariates W. For ease of exposition, and without loss of generality, we select the first k variables, *i.e.*, $A = (A_1, \ldots, A_k)$. Let $s \in \{0,1\}^k$ a binary vector of length k, and let $a(s) = (a_1(s_1), \ldots, a_k(s_k))$ indicate the joint specification of the k treatment levels. The joint baseline treatment level is denoted by $a(0) = (a_1(0), \ldots, a_k(0))$, and the joint target treatment level by $a(1) = (a_1(1), \ldots, a_k(1))$ where $a_i(s) \in \{0, 1, \ldots, t_i\}$. The k-point interaction quantifies the non-additive effect due to the joint change in treatment levels of the variables A from $a(0)$ to $a(1)$ relative to the sum of the effects of marginal changes for all subsets of variables with the remaining treatment variables held at their initial level. This parameter is a direct generalisation of the Average Treatment Effect (ATE), and defined in Beentjes and Khamseh (2020) as

$$\Psi^{(k)}_{a(0),a(1)}(P) = \sum_{s \in \{0,1\}^k} (-1)^{k-(s_1+\ldots+s_k)} \Psi_{a(s)}(P), \tag{1}$$

where $\Psi_{a(s)}(P)$ is the treatment-specific, covariate-adjusted outcome mean defined by

$$\begin{aligned} \Psi_{a(s)}(P) &= \mathbb{E}_P[\mathbb{E}_P(Y|A = a(s), W)] \\ &\equiv \mathbb{E}_P[\mathbb{E}_P(Y|A_1 = a_1(s_1), \ldots, A_k = a_k(s_k), W)]. \end{aligned} \tag{2}$$

Setting $k = 1$ yields a parameter quantifying the effect on Y of a single treatment variable A_1 changing from level $a(0) = a_1(0)$ to $a(1) = a_1(1)$, correcting for covariates W. This recovers the ATE,

$$\begin{aligned} \Psi^{(1)}_{a(0),a(1)} &= \sum_{s \in \{0,1\}} (-1)^{1-s} \Psi_{a(s)}(P) = \Psi_{a(1)}(P) - \Psi_{a(0)}(P) \\ &= \mathbb{E}_P[\mathbb{E}_P(Y|A = a(1), W) - \mathbb{E}_P(Y|A = a(0), W)]. \end{aligned}$$

Under additional causal assumptions $\Psi_{a(s)}(P)$ can be interpreted as the causal mean counterfactual outcome $\mathbb{E}[Y(a(s))]$ under the joint treatment assignment $A = a(s)$ where $s \in \{0,1\}^k$ (Rubin, 1974).

Before proceeding with the analysis of this target parameter, we recall some notation. As the parameter depends only on P through $Q = Q(P) = (\bar{Q}, Q_W)$, we sometimes write $\Psi(Q)$ instead of $\Psi(P)$. We denote the true outcome regression of Y on (A, W) by $\bar{Q}_0$, the true covariate distribution by $Q_{W,0}$, and abbreviate both by $Q(P_0) = Q_0 = (\bar{Q}_0, Q_{W,0})$. We write

$$g(a(s), w) = P(A = a(s) \mid W = w) = P(A_1 = a_1(s_1), \ldots, A_k = a_k(s_k) \mid W = w)$$

for the propensity score. Throughout, we assume that the true propensity score g_0 satisfies the positivity condition $\delta < g_0(a(s), w) < 1 - \delta$ for some $\delta > 0$, all covariates w in the support of $Q_{W,0}$, and all treatment assignments. Given a probability distribution $P \in \mathcal{M}_0$ and any P-integrable function f, we write $Pf = \mathbb{E}_P[f(O)] = \int f(o)dP(o)$. The empirical distribution on n variables $O_1, \ldots, O_n$ is denoted by $\mathbb{P}_n$, and the sample average of f with respect to $\mathbb{P}_n$ by $\mathbb{P}_n f = \frac{1}{n}\sum_{i=1}^n f(O_i)$. Henceforth, we leave the interaction order, as well as the initial and final treatment levels, implicit and write $\Psi(P)$ for the k-order interaction from levels $a(0)$ to $a(1)$ as defined in Equation (1).

Influence function and exact remainder

Recall that a regular estimator $\hat{\psi}_n$ of $\psi_0 = \Psi(Q_0)$ is asymptotically linear if it admits an expression of the form $\hat{\psi}_n = \psi_0 + \mathbb{P}_n D(P_0) + o_P(n^{-1/2})$, where $D(P_0)$ is a gradient of the statistical estimand Ψ at P_0 with respect to a statistical model $\mathcal{M}$. A function $D(P)(O)$ is a gradient of Ψ at P relative to $\mathcal{M}$ if

$$\frac{d}{d\epsilon}\Psi(P_\epsilon)\Big|_{\epsilon=0} = \int D(P)(o)s(o)dP(o) = \mathbb{E}_P\big[D(P)(O)s(O)\big], \tag{3}$$

for any regular one-dimensional parametric submodel $\{P_\epsilon\} \subseteq \mathcal{M}$ such that $P_{\epsilon=0} = P$ with mean-zero score function $s(o)$ at $\epsilon = 0$, and if $D(P) \in L^2_0(P)$. Here, for each $P \in \mathcal{M}$, we denote by $L^2_0(P)$ the Hilbert space of real-valued functions of O with zero mean, finite variance, defined on the support of P, and endowed with the covariance inner product given by $\langle f, g \rangle = \mathbb{E}_P[f(O)g(O)]$ for $f, g \in L^2_0(P)$. We denote the induced norm by $\| - \|_P$. The tangent space $\mathcal{T}_{\mathcal{M}}(P) \subseteq L^2_0(P)$ of $\mathcal{M}$ at P is the $L^2_0(P)$-closure generated by the score functions

of regular one-dimensional parametric models. In the nonparametric model $\mathcal{M}_0$, the scores generate the entire Hilbert space, *i.e.*, $\mathcal{T}_{\mathcal{M}}(P) = L_0^2(P)$ for every $P \in \mathcal{M}_0$. The squared $L^2(P)$-norm of the unique canonical gradient $D^*(P) \in \mathcal{T}_{\mathcal{M}}(P)$, also referred to as the efficient influence function (EIF), gives the generalized Cramer–Rao (CR) lower bound for estimating $\Psi(P)$ relative to $\mathcal{M}$ (Bickel et al., 1998). Under sampling from P_0, a regular asymptotically linear (RAL) estimator is efficient if and only if its influence function is the EIF. When $A = A_1$ is a single treatment variable, it is well known that the EIF of $\Psi_a(P)$ at P relative to $\mathcal{M}_0$ is

$$D_a^*(P)(O) = D_a^*(Q, g)(O) = \frac{\mathbb{1}\{A = a\}}{g(A, W)}\Big\{Y - \bar{Q}(A, W)\Big\} + \bar{Q}(a, W) - \Psi_a(Q),$$

where O is distributed according to $P \in \mathcal{M}_0$ (Van der Laan and Robins, 2003).

We verify that when $A = (A_1, \ldots, A_k)$ is a vector of treatments and $a(s) = (a_1(s_1), \ldots, a_k(s_k))$ denotes the joint treatment level specification of k treatments, the EIF of the parameter $\Psi_{a(s)}(P)$ has the same form.

Lemma 1 Let $k \geq 1$. The efficient influence function of the parameter $\Psi_{a(s)}(P)$ of Equation (2) is equal to

$$D_{a(s)}^*(P)(O) = D_{a(s)}^*(Q, g)(O) = \frac{\mathbb{1}\{A = a(s)\}}{g(A, W)}\Big\{Y - \bar{Q}(A, W)\Big\} + \bar{Q}(a(s), W) - \Psi_{a(s)}(Q). \tag{4}$$

Proof It suffices to check that $D_{a(s)}^*(P)$ is a gradient of $\Psi_{a(s)}(P)$ in the sense of Equation (3). To see this, let $\{P_\epsilon\} \subset \mathcal{M}$ be a regular submodel through $P_{\epsilon=0} = P$ with mean-zero score function

$$s(O) = \frac{d}{d\epsilon} \log dP_\epsilon(O)|_{\epsilon=0},$$

and consider its decomposition into partial score functions,

$$s(O) = s_Y(Y, A, W) + s_A(A, W) + s_W(W), \tag{5}$$

where, *e.g.*, $s_Y(Y, A, W) = \frac{d}{d\epsilon} \log dP_\epsilon(Y|A, W)|_{\epsilon=0} = s(O) - \mathbb{E}_P(s(O)|A, W)$. Note that $\mathbb{E}_P(s_Y(O)|A, W) = 0$. In particular, $\frac{d}{d\epsilon} dQ_{W,\epsilon}(W)|_{\epsilon=0} = s_W(W) dQ_W(W)$. We compute the parameter's pathwise derivative:

$$\begin{aligned}
\frac{d}{d\epsilon}\Psi_{a(s)}(P_\epsilon)\Big|_{\epsilon=0} &= \frac{d}{d\epsilon}\mathbb{E}_{P_\epsilon}[\mathbb{E}_{P_\epsilon}(Y|A = a(s), W)]\Big|_{\epsilon=0} \\
&= \int Y \frac{d}{d\epsilon}\Big|_{\epsilon=0} dP_\epsilon(Y|A = a(s), W) dQ_{W,\epsilon}(W) \\
&= \int Y \frac{d}{d\epsilon}\Big|_{\epsilon=0} dP_\epsilon(Y|A = a(s), W) dQ_W(W) + \int Y dP(Y|A = a(s), W) \frac{d}{d\epsilon}\Big|_{\epsilon=0} dQ_{W,\epsilon}(W),
\end{aligned}$$

where the second equality holds by the dominated convergence theorem. The first integral can be rewritten as

$$I_1 = \int Y \frac{\mathbb{1}\{A = a(s)\}}{g(A, W)} \frac{d}{d\epsilon}\Big|_{\epsilon=0} dP_\epsilon(Y|A, W) dP(A, W) = \int \frac{\mathbb{1}\{A = a(s)\}}{g(A, W)} Y s_Y(O) dP(O),$$

using the relations $\frac{d}{d\epsilon} dP_\epsilon(Y|A, W)|_{\epsilon=0} = s_Y(O) dP(Y|A, W)$ and $dP(O) = dP(Y|A, W) dP(A, W)$. Since $\mathbb{E}_P(s_Y(O)|A, W) = 0$ this integral is unchanged if we shift the integrand by a term of the form $f(A, W) s_Y(O)$ for some function $f(A, W) \in L_0^2(P)$. It follows that the first integral equals

$$I_1 = \int \frac{\mathbb{1}\{A = a(s)\}}{g(A, W)}\Big\{Y - \bar{Q}(A, W)\Big\} s_Y(O) dP(O).$$

Additionally, it is easy to check that the covariance inner product vanishes between any function $g(O) \in L_0^2(P)$ which has mean zero conditional on (A, W) and any function $f(A, W) \in L_0^2(P)$, *i.e.*, $\mathbb{E}_P[f(A, W) g(O)] = 0$. Since the term multiplying $s_Y(O)$ has mean zero conditional on (A, W), it follows that

$$I_1 = \int \frac{\mathbb{1}\{A = a(s)\}}{g(A, W)}\Big\{Y - \bar{Q}(A, W)\Big\} s(O) dP(O)$$

where we use that $s(O) - s_Y(O) = s_A(A, W) + s_W(W)$ is a function of the form $f(A, W) \in L_0^2(P)$ by Equation (5).

Next, the second integral can be rewritten as

$$I_2 = \int \mathbb{E}_P(Y|A = a(s), W)s_W(W)dQ_W(W) = \int \bar{Q}(a(s), W)s(O)dP(O),$$

using the relation $s_W(W) = \mathbb{E}_P[s(O)|W] - \mathbb{E}_P[s(O)] = \mathbb{E}_P[s(O)|W]$ since $s(O)$ is mean-zero. For the same reason, we have $\mathbb{E}_P[\Psi_{a(s)}(P)s(O)] = \Psi_{a(s)}(P)\mathbb{E}_P[s(O)] = 0$, and so the second integral equals

$$I_2 = \int \Big\{\bar{Q}(a(s), W) - \Psi_{a(s)}(P)\Big\}s(O)dP(O).$$

Collecting terms, we have now shown that $D^*_{a(s)}(P)$ is a gradient of $\Psi_{a(s)}(P)$:

$$\frac{d}{d\epsilon}\Psi_{a(s)}(P_\epsilon)\Big|_{\epsilon=0} = I_1 + I_2 = \int D^*_{a(s)}(P)(o)s(o)dP(o).$$

Since $\mathcal{T}_{\mathcal{M}}(P) = L_0^2(P)$ in the non-parametric model, we conclude that $D^*_{a(s)}(P)$ is the EIF of $\Psi_{a(s)}(P)$. □

The gradient $D^*(P_0)$ provides a first-order approximation of $\Psi(P)$ around the true value $\Psi(P_0)$ at the data-generating distribution P_0 via the Von Mises expansion of our pathwise differentiable parameter:

$$\Psi(P) - \Psi(P_0) = \big(P - P_0\big)D^*(P) + R(P, P_0). \tag{6}$$

This equation defines the second-order exact remainder $R(P, P_0) = \Psi(P) - \Psi(P_0) + P_0D^*(P)$, where we have used $PD^*(P) = 0$ since $D^*(P) \in L_0^2(P)$. When $A = A_1$ is a single treatment variable, it is well known that the second-order exact remainder of the parameter $\Psi_a(P)$ at P relative to $\mathcal{M}_0$ can be written as

$$R_a(P, P_0) = R_a\big(Q_a, Q_{a,0}, g_a, g_{a,0}\big) = P_0\Big\{\big(\bar{Q}_a - \bar{Q}_{a,0}\big)\big(g_a - g_{a,0}\big)/g_a\Big\}$$

where we write $\bar{Q}_a(W) = \bar{Q}(a, W)$, $g_a(W) = P(A = a|W)$ and their analogues implied by P_0. In particular, the remainder of this parameter is double robust by the Cauchy–Schwarz inequality:

$$|R_a(P, P_0)| \leq \|\bar{Q}_{a,0} - \bar{Q}_a\|_{P_0} \cdot \|(g_{a,0} - g_a)/g_a\|_{P_0}. \tag{7}$$

We now verify that the second-order exact remainder of the parameter $\Psi_{a(s)}(P)$ has the same form for a vector of treatments $A = (A_1, \ldots, A_k)$ and a joint treatment level specification $a(s) = (a_1(s_1), \ldots, a_k(s_k))$.

Lemma 2 Let $k \geq 1$. The second-order exact remainder of the parameter $\Psi_{a(s)}(P)$ of Equation (2) equals

$$R_{a(s)}(P, P_0) = P_0\Big\{\big(\bar{Q}_{a(s)} - \bar{Q}_{a(s),0}\big)\big(g_{a(s)} - g_{a(s),0}\big)/g_{a(s)}\Big\}. \tag{8}$$

Proof Combining the definition of $\Psi_{a(s)}(P)$ with its EIF of Equation (4), we see that

$$\begin{aligned} R_{a(s)}(P, P_0) &\equiv \Psi_{a(s)}(P) - \Psi_{a(s)}(P_0) + P_0D^*_{a(s)}(P) \\ &= P_0\left\{\frac{\mathbb{1}\{A = a(s)\}}{g(A, W)}\big[Y - \bar{Q}(A, W)\big] + \bar{Q}(a(s), W) - \bar{Q}_0(a(s), W)\right\} \end{aligned} \tag{9}$$

where we have written $\Psi_{a(s)}(P_0) = \mathbb{E}_{P_0}\big[\bar{Q}_0(a(s), W)\big]$. Decomposing the first half of this expression yields

$$I_1 = P_0\left\{\frac{\mathbb{1}\{A = a(s)\}}{g(A, W)}\big[Y - \bar{Q}_0(A, W)\big]\right\} + P_0\left\{\frac{\mathbb{1}\{A = a(s)\}}{g(A, W)}\big[\bar{Q}_0(A, W) - \bar{Q}(A, W)\big]\right\}.$$

The first term in I_1 vanishes by the tower rule since $P_0\big\{Y - \bar{Q}_0(A, W) \mid A, W\big\} = \bar{Q}_0(A, W) - \bar{Q}_0(A, W) = 0$. Another application of the tower rule allows us to rewrite the second term in I_1 as

$$\begin{aligned} P_0\left\{\frac{\mathbb{1}\{A = a(s)\}}{g(A, W)}\big[\bar{Q}_0(A, W) - \bar{Q}(A, W)\big]\right\} &= P_0\left\{\frac{P_0\big[\mathbb{1}\{A = a(s)\}|W\big]}{g(a(s), W)}\big[\bar{Q}_0(a(s), W) - \bar{Q}(a(s), W)\big]\right\} \\ &= P_0\left\{\frac{g_0(a(s), W)}{g(a(s), W)}\big[\bar{Q}_0(a(s), W) - \bar{Q}(a(s), W)\big]\right\}. \end{aligned}$$

We find $I_1 = P_0\big\{\big(\bar{Q}_{a(s)} - \bar{Q}_{a(s),0}\big)\big(-g_{a(s),0}/g_{a(s)}\big)\big\}$. Finally, we write the second term in Equation (9) as

$$I_2 = P_0\left\{\bar{Q}(a(s), W) - \bar{Q}_0(a(s), W)\right\} = P_0\left\{\big(\bar{Q}_{a(s)} - \bar{Q}_{a(s),0}\big)\big(g_{a(s)}/g_{a(s)}\big)\right\}.$$

It follows that $R_{a(s)}(P, P_0) = I_1 + I_2 = P_0\big\{\big(\bar{Q}_{a(s)} - \bar{Q}_{a(s),0}\big)\big(g_{a(s)} - g_{a(s),0}\big)/g_{a(s)}\big\}$ as claimed. □

Since the k-point interaction parameter is a linear combination of parameters $\Psi_{a(s)}(P)$ as in Equation (2), its EIF and second-order remainder are linear combinations of the EIF and second-order remainder of $\Psi_{a(s)}(P)$ respectively. Using the results of Lemmas 1 and 2 above, we now formally derive the EIF and second-order exact remainder of our k-point interaction parameter $\Psi_{a(0),a(1)}(P)$, thus proving Proposition 1.

Proposition The canonical gradient (or EIF) and second-order exact remainder of the k-point interaction target parameter $\Psi^{(k)}_{a(0),a(1)}(P)$ of Equation (1) at P relative to the non-parametric model $\mathcal{M}_0$ are equal to

$$D^*_{a(0),a(1)}(P) = \sum_{s\in\{0,1\}^k} (-1)^{k-(s_1+\ldots+s_k)} D^*_{a(s)}(P) \tag{10}$$

$$R_{a(0),a(1)}(P, P_0) = \sum_{s\in\{0,1\}^k} (-1)^{k-(s_1+\ldots+s_k)} R_{a(s)}(P, P_0), \tag{11}$$

respectively, where the expressions of $D^*_{a(s)}(P)$ and $R_{a(s)}(P, P_0)$ are given in Equations (4) and (8).

Proof By its definition in Equation (3), it follows that the gradient of a linear combination of pathwise differentiable parameters is the linear combination of their gradients. By Lemma 1, we deduce $D^*_{a(0),a(1)}(P)$ as in Equation (10). Reorganising the Von Mises expansion of Equation (6) as

$$R_{a(0),a(1)}(P, P_0) = \Psi_{a(0),a(1)}(P) - \Psi_{a(0),a(1)}(P_0) + P_0 D^*_{a(0),a(1)}(P)$$

shows that the second-order exact remainder is similarly linear in the target parameter because so are both the gradient and its defining equation. We immediately obtain the second-order exact remainder of our k-point interaction parameter as in Equation (11). This completes the proof. □

As a corollary, we deduce the double robustness of our interaction parameter from Equation (8) by combining the triangle inequality with Equation (7) which follows from the Cauchy–Schwarz inequality in $L^2_0(P)$:

$$\begin{aligned} |R_{a(0),a(1)}(P, P_0)| &= \Big|\sum_{s\in\{0,1\}^k} (-1)^{k-(s_1+\ldots+s_k)} R_{a(s)}(P, P_0)\Big| \\ &\le \sum_{s\in\{0,1\}^k} |R_{a(s)}(P, P_0)| \\ &\le \sum_{s\in\{0,1\}^k} \|\bar{Q}_{a(s),0} - \bar{Q}_{a(s)}\|_{P_0} \cdot \|(g_{a(s),0} - g_{a(s)})/g_{a(s)}\|_{P_0}, \end{aligned} \tag{12}$$

where the first equality follows from Equation (11) in the Proposition, the second inequality is the triangle inequality in $L^2_0(P)$, and the third and final inequality is the Cauchy–Schwarz inequality of Equation (7).

Semi-parametric efficient estimators

Let $\hat{P}_n$ be an estimator of P_0 based on the available data $\mathbb{P}_n$, and denote by $\hat{\psi}_n \equiv \Psi(\hat{P}_n)$ the corresponding plug-in estimator. We use the EIF and Von Mises expansion to construct non-parametric efficient estimators of interaction via (i) one-step bias correction (Pfanzagl and Wefelmeyer, 1985), and (ii) targeted minimum

loss-based estimation (TMLE) (Van der Laan and Rubin, 2006; Van der Laan and Rose, 2011, 2018). Since Ψ is path-wise differentiable, we inspect its Von Mises expansion:

$$\Psi(\hat{P}_n) - \Psi(P_0) = \big(\hat{P}_n - P_0\big)D^*(\hat{P}_n) + R(\hat{P}_n, P_0).$$

By adding and subtracting terms, this can be rewritten as

$$\Psi(\hat{P}_n) - \Psi(P_0) = \big(\mathbb{P}_n - P_0\big)D^*(\hat{P}) - \mathbb{P}_n D^*(\hat{P}_n) + \big(\mathbb{P}_n - P_0\big)\Big\{D^*(\hat{P}_n) - D^*(\hat{P})\Big\} + R(\hat{P}_n, P_0)$$

where $\hat{P}$ denotes the in-probability limit of $\hat{P}_n$, and we have used $PD^*(P) = 0$ for any $P \in \mathcal{M}_0$. Let $\bar{Q}_n$ and g_n be the corresponding components of $\hat{P}_n$, let $\bar{Q}$ and g be their in-probability limits (components of $\hat{P}$), and let $\bar{Q}_0$ and g_0 be the components of P_0. Following Benkeser et al. (2017), we write the above equation as

$$\Psi(Q_n) - \Psi(Q_0) = \big(\mathbb{P}_n - P_0\big)D^*(Q, g) - B_n(Q_n, g_n) + M_n(Q_n, Q, g_n, g) + R(Q_n, Q_0, g_n, g_0) \quad (13)$$

where the two additional terms are the first-order bias term, $B_n(Q_n, g_n) = \mathbb{P}_n D^*(Q_n, g_n)$, and an empirical process term $M_n(Q_n, Q, g_n, g) = \big(\mathbb{P}_n - P_0\big)\Big\{D^*(Q_n, g_n) - D^*(Q, g)\Big\}$. As discussed in the main manuscript, it suffices to focus on the bias term $B_n(Q_n, g_n)$ of the k-order interaction parameter of Equation (1):

$$B_n(Q_n, g_n) = \frac{1}{n}\sum_{i=1}^{n}\left[\sum_{s\in\{0,1\}^k}(-1)^{k-(s_1+\ldots+s_k)}\frac{\mathbb{1}\{a_i = a(s)\}}{g_n(a_i, w_i)}\right]\Big\{y_i - \bar{Q}_n(a_i, w_i)\Big\}, \quad (14)$$

where $o_i = (y_i, a_i, w_i) = (y_i, a_{j_1,i}, \ldots, a_{j_k,i}, w_i)$ is the ith observed data point. In practice, this term can be substantial. We discuss two general strategies to deal with the first-order bias term $B_n(Q_n, g_n)$ and obtain an asymptotically linear estimator of the k-order interaction parameter, provided the rate conditions for the empirical process term and second-order exact remainder are met. The empirical process term is of the required rate $o_P(n^{-1/2})$ when $D^*(Q_n, g_n)$ belongs to a P_0-Donsker class with probability tending to one and $P_0\big\{D^*(Q_n, g_n) - D^*(Q, g)\big\}^2$ converges to zero in probability (Benkeser et al., 2017), or by employing sample splitting. Throughout, we refer to these as the *canonical* and the *cross-validated* (CV) approaches.

Canonical estimators

The One-Step Estimator (OSE) of Pfanzagl and Wefelmeyer (1985) is an infinite-dimensional generalisation of the Newton-Raphson method in which the first-order bias term is added to the plug-in estimator,

$$\hat{\psi}_n^+ := \hat{\psi}_n + B_n(Q_n, g_n). \quad (15)$$

Provided the rate conditions on M_n and R_n hold, Equation (13) reads

$$\hat{\psi}_n^+ - \psi_0 = \mathbb{P}_n D^*(Q, g) + o_P(n^{-1/2}) \quad (16)$$

demonstrating that the OSE is asymptotically linear with variance equal to the non-parametric efficiency bound given by the variance of the EIF $D^*(Q, g)$ provided $Q = Q_0$ and $g = g_0$. For a description of sampling behaviour when either $Q = Q_0$ or $g = g_0$, see Section 2.2 of Benkeser et al. (2017). For the average treatment effect, *i.e.*, our target parameter with $k = 1$, the one-step estimator reduces to the Augmented Inverse Propensity Weighting (AIPW) estimator introduced by Robins et al. (1994). While the one-step estimator is asymptotically efficient and straightforward to implement, its finite-sample performance can suffer since it is not a plug-in estimator and the first-order bias term may push the estimate outside of the target parameter's natural range.

The second approach, introduced by Van der Laan and Rubin (2006), instead updates the fit Q_n of Q_0 in an iterative procedure to a final fit Q_n^* such that $B_n(Q_n^*, g_n) = 0$. This updating step yields the Targeted Maximum-Likelihood, or Targeted Minimum Loss-based, Estimator (TMLE),

$$\hat{\psi}_n^{\text{tmle}} := \Psi(Q_n^*). \quad (17)$$

Similar to OSE, provided the rate conditions on M_n and R_n hold, Equation (13) reads

$$\hat{\psi}_n^{\text{tmle}} - \psi_0 = \mathbb{P}_n D^*(Q, g) + o_P(n^{-1/2}) \quad (18)$$

demonstrating that the TMLE is asymptotically linear with variance equal to the non-parametric efficiency bound given by the variance of the EIF $D^*(Q, g)$ when $Q = Q_0$ and $g = g_0$. The sampling behaviour when either $Q = Q_0$ or $g = g_0$ is described in Section 2.2 of Benkeser et al. (2017). TMLE is a plug-in estimator and hence enjoys finite-sample robustness properties (Porter et al., 2011).

TMLE updating step

We discuss the TMLE updating step implemented in our package `TMLE.jl` and software `TarGene`. It consists in a linear regression (for continuous outcome Y) or logistic regression (for binary outcome Y) with offset, the initial fit $\bar{Q}_n(a,w)$, and covariate, the so-called clever covariate $H(g)(a,w)$ derived from the EIF $D^*(Q,g)$:

$$\begin{aligned} \bar{Q}_{n,\epsilon}(a,w) &= \bar{Q}_n(a,w) + \epsilon H(g)(a,w), \\ \operatorname{logit} \bar{Q}_{n,\epsilon}(a,w) &= \operatorname{logit} \bar{Q}_n(a,w) + \epsilon H(g)(a,w) \end{aligned} \tag{19}$$

respectively. The parameter ϵ is fitted by minimum loss-based estimation,

$$\hat{\epsilon} = \arg\min_{\epsilon} \mathbb{P}_n \mathcal{L}\{\bar{Q}_{n,\epsilon}\}, \tag{20}$$

with respect to the squared loss function $\mathcal{L}\{f\}(O) = \{f(W,A) - Y\}^2$ for continuous outcome Y or the log-loss function $\mathcal{L}\{f\}(O) = -Y \log f(W,A) - (1-Y)\log\big(1 - f(A,W)\big)$ for binary outcome Y, where f is a function of (W,A). The targeted update $\bar{Q}_n^* \equiv \bar{Q}_{n,\hat{\epsilon}}$ defines the TMLE of Equation (17), where we use the empirical distribution $\mathbb{Q}_W$ of Q_W which is not updated as it is the nonparametric MLE. These fluctuations, loss functions, and clever covariate are chosen so that the update $Q_n^* = (\bar{Q}_n^*, \mathbb{Q}_W)$ solves the EIF, *i.e.*, it eliminates the first-order bias $B_n(Q_n^*, g_n) = 0$. To see this, note

$$\frac{d}{d\epsilon}\mathcal{L}\{\bar{Q}_{n,\epsilon}\}(O)\Big|_{\epsilon=0} = H(g)(A,W)\{Y - \bar{Q}_n(A,W)\}. \tag{21}$$

Since $\hat{\epsilon}$ is the minimiser of the empirical loss $\mathbb{P}_n\mathcal{L}\{\bar{Q}_{n,\epsilon}\}$ and $\bar{Q}_n^* \equiv \bar{Q}_{n,\hat{\epsilon}}$, it follows that

$$0 = \frac{d}{d\epsilon}\mathbb{P}_n\mathcal{L}\{\bar{Q}_{n,\epsilon}^*\}\Big|_{\epsilon=0} = \mathbb{P}_n\Big\{H(g_n)(A,W)\{Y - \bar{Q}_n^*(A,W)\}\Big\} \equiv B_n(Q_n^*, g_n) \tag{22}$$

by Equation (14). Here $\bar{Q}_{n,\epsilon}^*$ denotes the corresponding fluctuation of Equation (19) but with offset $\bar{Q}_n^*$ so that $\bar{Q}_{n,\epsilon=0}^* \equiv \bar{Q}_n^*$. Thus, for the k-point interaction $A\colon a(0) \to a(1)$ the clever covariate should be set to

$$H(g_n)(A,W) = \sum_{s\in\{0,1\}^k} (-1)^{k-(s_1+\ldots+s_k)} \frac{\mathbb{1}\{A = a(s)\}}{g_n(A,W)}, \tag{23}$$

generalising the clever covariate for the average treatment effect, *i.e.*, interaction with $k=1$. Alternatively, the clever covariate can be obtained as the Riesz representer of the target parameter (Lee and Schuler, 2025). This completely defines $\hat{\psi}_n^{\text{tmle}}$ of Equation (17) and demonstrates it is asymptotically linear as per Equation (18). Note that we use a single regression parameter ϵ to update the interaction estimator itself, via the clever covariate in Equation (23), rather than each individual conditional expectation value $\mathbb{E}[\bar{Q}_n(a(s),W)]$.

Weighted TMLE updating step

In finite samples, it has been observed in simulations (Sofrygin and Van der Laan, 2017) that performance in terms of bias, variance, and coverage increases when fitting ϵ in Equation (19) via weighted regression, *i.e.*, by minimising a weighted version of the empirical loss of Equation (20). Specifically, performance improves in the presence of near-positivity violations when the estimated propensity score $g_n(A,W)$ is close to zero. This is particularly relevant in fields such as genetics, where DNA variants may be rare. The weighted approach places the term $1/g_n(A,W)$ in the loss function,

$$\mathcal{L}_{g_n}\{f\}(O) \equiv \frac{1}{g_n(A,W)}\mathcal{L}\{f\}(O),$$

and removes it from the clever covariate. The corresponding weighted fluctuations are

$$\begin{aligned} \bar{Q}_{n,\epsilon}^{(w)}(a,w) &= \bar{Q}_n(a,w) + \epsilon H'(a,w), \\ \operatorname{logit} \bar{Q}_{n,\epsilon}^{(w)}(a,w) &= \operatorname{logit} \bar{Q}_n(a,w) + \epsilon H'(a,w) \end{aligned} \tag{24}$$

where the weighted clever covariate of our k-point interaction parameter $\Psi_{a(0),a(1)}(P)$ of Equation (1) is

$$H'(A,W) = \sum_{s\in\{0,1\}^k} (-1)^{k-(s_1+\ldots+s_k)}\mathbb{1}\{A = a(s)\}. \tag{25}$$

The one-dimensional parameter $\hat{\epsilon}$ is now fitted by minimising the empirical weighted loss,

$$\hat{\epsilon} = \arg\min_{\epsilon} \mathbb{P}_n \mathcal{L}_{g_n}\{\bar{Q}_{n,\epsilon}\},$$

yielding $\bar{Q}_n^* \equiv \bar{Q}_{n,\hat{\epsilon}}^{(w)}$ and $Q_n^* = (\bar{Q}_n^*, \mathbb{Q}_W)$. The weighted TMLE (wTMLE) is defined as $\hat{\psi}_n^{\mathrm{wtmle}} \equiv \Psi(Q_n^*)$.

We show that applying the (weighted) TMLE update step solves the EIF, *i.e.*, it satisfies $B_n(Q_n^*, g_n) = 0$. The update step can be performed in two different ways: (i) by *maximising* the log-likelihood of the fluctuation $Q_{n,\epsilon}$ through the initial fit Q_n, or (ii) by *minimising* an appropriately chosen loss function $\mathcal{L}(Q_{n,\epsilon})$ of the fluctuation $Q_{n,\epsilon}$ through the initial fit Q_n as in Equations (19) and (24). The former approach is called targeted maximum likelihood estimation whereas the latter is called targeted minimum loss-based estimation; both are abbreviated TMLE. In `TMLE.jl`, we apply the loss-based approach to estimate the k-point interaction parameter with clever covariate as in Equation (23) or, alternatively, weighted clever covariate as in Equation (25).

Proposition 3 Let Y be a binary outcome, let f be a function of the data O taking values in the unit interval $[0, 1]$, and consider the log-loss function

$$\mathcal{L}[f](O) = -\log\Big\{f(O)^Y [1 - f(O)]^{1-Y}\Big\}.$$

Applying TMLE by (iteratively) minimising the log-loss function $\mathcal{L}[Q_n^k(\epsilon)]$ of the fluctuation $Q_n^k(\epsilon)$ with respect to ϵ given by the logistic regression

$$Q_n^k(\epsilon) = \mathrm{expit}\{\mathrm{logit}\, Q_n^k + \epsilon H(g_n)\}, \quad \text{where} \quad k \geq 0,$$

using $\hat{\epsilon} = \arg\min_\epsilon \sum_i^n \mathcal{L}\big[Q_n^k(\epsilon)\big](o_i)$ to define $Q_n^{k+1} \equiv Q_n^k(\hat{\epsilon})$, solves the empirical IF.

Similarly, let g_n be the estimated propensity score, and consider the g_n-weighted log-loss function

$$\mathcal{L}_{g_n}[f](O) = -\frac{1}{g_n(O)} \log\Big\{f(O)^Y [1 - f(O)]^{1-Y}\Big\}.$$

Applying wTMLE by (iteratively) minimising the weighted log-loss function $\mathcal{L}_{g_n}[\tilde{Q}_n^k(\epsilon)]$ of the weighted fluctuation $\tilde{Q}_n^k(\epsilon)$ with respect to ϵ given by the logistic regression

$$\tilde{Q}_n^k(\epsilon) = \mathrm{expit}\{\mathrm{logit}\, \tilde{Q}_n^k + \epsilon H'(g_n)\}, \quad \text{where} \quad \tilde{Q}_n^0 = Q_n^0,\ k \geq 0,$$

using $\tilde{\epsilon} = \arg\min_\epsilon \sum_i^n \mathcal{L}_{g_n}\big[\tilde{Q}_n^k(\epsilon)\big](o_i)$ to define $\tilde{Q}_n^{k+1} \equiv \tilde{Q}_n^k(\tilde{\epsilon})$, solves the empirical IF.

Proof To see this, recall that $\mathrm{expit}(x) = \{1+\exp(-x)\}^{-1}$ is the inverse of the function $\mathrm{logit}(p) = \log\{p/(1-p)\}$ for $p \in (0, 1)$, and for any $a, b \in \mathbb{R}$ we have the relation

$$\frac{d}{d\epsilon}\Big|_{\epsilon=0} \mathrm{expit}(a + b\epsilon) = b\, \mathrm{expit}(a)\big\{1 - \mathrm{expit}(a)\big\} \tag{26}$$

as is easily checked. First, we minimise the (unweighted) log-loss function:

$$\frac{d}{d\epsilon}\Big|_{\epsilon=0} \mathcal{L}\big[Q_n^k(\epsilon)\big] = \left\{\frac{1-Y}{1-Q_n^k} - \frac{Y}{Q_n^k}\right\} \frac{d}{d\epsilon} Q_n^k(\epsilon)\Big|_{\epsilon=0}.$$

In order to evaluate this expression, we use the result of Equation (26) to compute

$$\frac{d}{d\epsilon} Q_n^k(\epsilon)\Big|_{\epsilon=0} = H(g_n) Q_n^k\big(1 - Q_n^k\big).$$

Putting both computations together, we conclude

$$\frac{d}{d\epsilon}\Big|_{\epsilon=0} \mathcal{L}\big[Q_n^k(\epsilon)\big] = H(g_n)\Big\{(1-Y)Q_n^k - Y\big(1 - Q_n^k\big)\Big\} = -H(g_n)\big(Y - Q_n^k\big)$$

which is the first component of the EIF of the interaction parameter. Thus if Q_n^k has been updated to the final Q_n^* such that the derivative with respect to ϵ of the empirical mean of the log-loss function applied to a

further fluctuation $Q_n^*(\epsilon)$ vanishes, we have

$$0 = \frac{d}{d\epsilon}\Big|_{\epsilon=0} \sum_{i=1}^{n} \mathcal{L}\big[Q_n^*(\epsilon)\big](o_i) = -\sum_{i=1}^{n} H(g_n)(o_i)\big\{y_i - Q_n^*(o_i)\big\}.$$

This means that the empirical mean of the EIF, *i.e.*, the first-order bias, vanishes as required.

Next, using similar arguments, we minimise the g_n-weighted log-loss function:

$$\frac{d}{d\epsilon}\Big|_{\epsilon=0} \mathcal{L}_{g_n}\big[\tilde{Q}_n^k(\epsilon)\big] = \frac{1}{g_n}\left\{\frac{1-Y}{1-\tilde{Q}_n^k} - \frac{Y}{\tilde{Q}_n^k}\right\} \frac{d}{d\epsilon}\tilde{Q}_n^k(\epsilon)\Big|_{\epsilon=0}.$$

In order to evaluate this expression, we again use the result of Equation (26) to compute

$$\frac{d}{d\epsilon}\tilde{Q}_n^k(\epsilon)\Big|_{\epsilon=0} = H'(g_n)\tilde{Q}_n^k\big(1-\tilde{Q}_n^k\big).$$

Putting both computations together, noting that $H'(g_n)/g_n = H(g_n)$, we conclude

$$\frac{d}{d\epsilon}\Big|_{\epsilon=0} \mathcal{L}_{g_n}\big[\tilde{Q}_n^k(\epsilon)\big] = H(g_n)\Big\{(1-Y)\tilde{Q}_n^k - Y\big(1-\tilde{Q}_n^k\big)\Big\} = -H(g_n)\big(Y - \tilde{Q}_n^k\big)$$

and we conclude by the same argument as for the (unweighted) log-loss function. □

By a similar argument, (w)TMLE solves the EIF for our interaction parameter with a continuous outcome Y. In this case, one combines the squared-loss function $\mathcal{L}[f](O) = \big\{Y - f(O)\big\}^2$ with the fluctuation given by the linear regression $Q_n^0(\epsilon) = Q_n^0 + \epsilon H(g_n)$ of Equation (19) where $H(g_n)$ is the clever covariate as above. For wTMLE, one combines the g_n-weighted squared-loss function

$$\mathcal{L}_{g_n}[f](O) = \frac{1}{g_n(O)}\big\{Y - f(O)\big\}^2$$

with the fluctuation given by the linear regression $\tilde{Q}_n^0(\epsilon) = Q_n^0 + \epsilon H'(g_n)$ of Equation (24).

Cross-validated estimators

Estimating the functions (Q_n, g_n) with data-adaptive or ML algorithms whilst evaluating OSE and (w)TMLE on the same dataset may lead to decreased performance of the canonical estimators as it can affect the required rate conditions of the empirical process term M_n. By using cross-validated (or sample-splitting) versions of these estimators, we can allow for complex algorithms to fit (Q_n, g_n) on part of the data whilst maintaining performance of OSE and (w)TMLE by evaluating these estimators on a held-out part of the data, essentially treating the fits of (Q_n, g_n) as fixed.[1]

More precisely, for K-fold sample splitting we split the data into $K \geq 2$ disjoint folds. We write $k(i) \in \{1, 2, \ldots, K\}$ for the fold to which sample i belongs, and $-k(i)$ for the union of the $K-1$ remaining folds. Similarly, we write Q_n^k for an estimator of $Q_0 = (\bar{Q}_0, Q_W)$ using the samples in fold k only, and we write Q_n^{-k} for an estimator of Q_0 using the samples in all folds but k. We use this notation to define the cross-validated versions of OSE and (w)TMLE. The CV-OSE is

$$\hat{\psi}_n^{\text{cv},+} := \sum_{k=1}^{K} \frac{n_k}{n}\Big\{\Psi\left(\bar{Q}_n^{-k}, \mathbb{Q}_W^k\right) + \mathbb{P}_n^k D^*\left(Q_n^{-k}, g_n^{-k}\right)\Big\}, \tag{27}$$

where $n_k = |\{i\colon k(i) = k\}|$ denotes the number of samples in the ith fold, and $\mathbb{P}_n^k$ denotes the empirical distribution of the kth fold. Importantly, for the K estimators in the sum, averages are taken over fold k (via $\mathbb{Q}_W^k$ and $\mathbb{P}_n^k$) whereas Q_n^{-k} and g_n^{-k} are estimated on all other folds.

[1] Specifically, using sample-splitting avoids the P_0-Donsker condition on algorithms used to fit Q_n and g_n.

For the CV-TMLE, the parameter $\hat{\epsilon}$ is defined in a pooled manner by the objective

$$\hat{\epsilon} = \arg\min_{\epsilon} \sum_{k=1}^{K} \mathbb{P}_n^k \mathcal{L}\{\bar{Q}_{n,\epsilon}^{-k}\} = \arg\min_{\epsilon} \sum_{k=1}^{K} \sum_{i:k(i)=k} \mathcal{L}\{\bar{Q}_{n,\epsilon}^{-k}\}(O_i) \tag{28}$$

where $\bar{Q}_{n,\epsilon}^{-k}$ is the respective path estimated on the folds in $-k$ and $\mathcal{L}$ is the respective loss function for continuous and binary outcomes. Note that the loss of a sample i in fold $k(i) = k$ is computed relative to nuisance functions fitted on all other folds $-k$. The targeted update step now satisfies

$$\bar{Q}_n^*(O_i) = \bar{Q}_{n,\hat{\epsilon}}^{-k(i)}(O_i) \tag{29}$$

for all samples $i = 1, 2, \ldots, n$, and depends on the pooled estimate $\hat{\epsilon}$. The CV-TMLE is defined as

$$\hat{\psi}_n^{\text{cv,tmle}} := \sum_{k=1}^{K} \frac{n_k}{n} \Psi\left(\bar{Q}_n^*, \mathbb{Q}_W^k\right). \tag{30}$$

While no longer a substitution estimator, the CV-TMLE respects the natural bounds of the target parameter because it is an average of substitution estimators. The weighted CV-TMLE, which we denote by $\hat{\psi}_n^{\text{cv,wtmle}}$, is defined analogously by using the weighted fluctuation and weighted loss function.

Variance estimators

We obtain asymptotic Wald-type confidence intervals and hypothesis tests for interaction using the asymptotic normality of the interaction estimators constructed in the previous section. If $\hat{\psi}_n$ is any of these estimators, with estimated nuisance functions (Q_n, g_n), then

$$\sqrt{n}\big(\hat{\psi}_n - \psi_0\big) = \sqrt{n}\,\mathbb{P}_n D^*(Q, g) + o_P(1) \rightsquigarrow \mathcal{N}\big(0, \operatorname{Var} D^*(Q, g)\big)$$

by the Central Limit Theorem. Here, recall that (Q, g) are the in-probability limits of (Q_n, g_n) respectively, and we assume either $Q = Q_0$ or $g = g_0$ (or both). In practice, for the canonical estimators of Equations (15) and (17), we use the sample variance estimator $\hat{\sigma}_n^2$ built from (Q_n, g_n) to approximate the variance of the EIF:

$$\hat{\sigma}_n^2 = \mathbb{P}_n\{D^*(Q_n, g_n)\}^2 = \frac{1}{n} \sum_{i=1}^{n} \Big\{D^*(Q_n, g_n)(o_i)\Big\}^2. \tag{31}$$

For the cross-validated versions of the OSE and (weighted) TMLE of Equations (27) and (30), we approximate the variance of the EIF with cross-validated sample variance estimators $\hat{\sigma}_{\text{cv},n}^2$ built from the nuisance functions (Q_n^{-k}, g_n^{-k}) estimated on all folds but k for $k = 1, \ldots, K$, namely

$$\hat{\sigma}_{\text{cv},n}^2 = \sum_{k=1}^{K} \frac{n_k}{n} \mathbb{P}_n^k \{D^*\left(Q_n^{-k}, g_n^{-k}\right)\}^2 = \frac{1}{n} \sum_{k=1}^{K} \sum_{i:k(i)=k} \Big\{D^*\left(Q_n^{-k}, g_n^{-k}\right)(o_i)\Big\}^2. \tag{32}$$

Both $\hat{\sigma}_n^2$ and $\hat{\sigma}_{\text{cv},n}^2$ are consistent estimators of $\operatorname{Var} D^*(Q_0, g_0)$ if the rate conditions on M_n and R_n hold. We obtain asymptotically valid $(1-\alpha) \times 100\%$ Wald-type confidence intervals of the form

$$\widehat{\text{CI}}_n = \left(\hat{\psi}_n - z_{1-\alpha/2}\frac{\hat{\sigma}_n}{\sqrt{n}}, \hat{\psi}_n + z_{1-\alpha/2}\frac{\hat{\sigma}_n}{\sqrt{n}}\right), \tag{33}$$

where z_β denotes the β-quantile of the standard normal distribution. Similarly, under the null hypothesis of no interaction, $H_0\colon \psi_0 = 0$, we can use $\sqrt{n}\hat{\psi}_n/\hat{\sigma}_n \sim \mathcal{N}(0,1)$ to test for interaction.

SUPPLEMENT TO SIMULATIONS

Variable Selection

For each genetic variant A_j, we assume the set of parent variables consists of the first six principal components derived from the genotyping data. While limitations of PCA have been pointed out (Elhaik, 2022), it is still standard practice to use PCA to adjust for genetic ancestry (Price et al., 2006; Mbatchou et al., 2021; Yang et al., 2011; Loh et al., 2015).

In principle, variation across the entire genome as well as environmental variables could be causal of a given trait's variation. To restrict the dimensionality of the problem we only consider a small subset of these putative causes. Environmental variables are kept to the standard covariates, namely, age at assessment and genetic sex. To include potential causal variants from the genome, we use published GWAS results from GeneATLAS (Canela-Xandri et al., 2018). More precisely, we select a maximum of 50 variants from all variants associated with the outcome of interest (p-value $< 10^{-5}$). Furthermore, we require these variants to (i) be at least one million base pairs away from each other to avoid linkage disequilibrium (LD), and (ii) have a minor allele frequency of at least 0.01. In Figure 1, these selected variants and environmental variables are contained within the C variable. The fact that selected variants are not generated from principal components greatly reduces computational burden but poses a mild limitation. Since all variables in C are jointly sampled, the dependence structure within selected variants is preserved. However, selected variants and variants used as treatment to define the causal estimands, are independent in this simulation. In a more realistic scenario they would only be independent once conditioned on principal components.

Algorithm 1 Sieve Neural Network Estimator

```
procedure SNNE(hiddenLayerSizes, dataset, maxSievePatience)
    trainingSet, validationSet ← split(dataset)
    bestModel ← build(hiddenLayerSizes[1])
    bestValidationLoss ← train(bestModel, trainingSet, validationSet)
    sievePatience ← 0
    for hiddenLayerSize ∈ hiddenLayerSizes[2 : end] do
        model ← build(hiddenLayerSize)
        validationLoss ← train(model, trainingSet, validationSet)
        if validationLoss ≤ bestValidationLoss then
            bestValidationLoss ← validationLoss
            bestModel ← model
            sievePatience ← 0
        else
            sievePatience ← sievePatience + 1
        end if
        if sievePatience == maxSievePatience then
            break
        end if
    end for
    return bestModel
end procedure
```

Model Selection

The second requirement for the simulation to be realistic is that the density estimators should capture complex patterns, which means the model class must be large. Neural networks, have been shown to be able to approximate a large class of function and scale seamlessly to large datasets such as the UK Biobank Hornik et al. (1989). We thus used two types of neural networks depending on the type of the density's outcome variable. For categorical variables, including binary outcomes, a one hidden-layer perceptron was employed, while for continuous variables we used a one layer mixture density network Haykin (1998); Bishop (1994). In essence, the models were designed to be computationally efficient for fast training while remaining flexible enough to capture complex interactions between variables. In all cases, the size of the hidden layer was chosen via cross-validation, using a sieve Chen (2007). That is, the size of the model was chosen data adaptively by sequentially increasing the hidden layer size (candidates: $[5, 10, 20, 40, 60, 80, 100, 120, 140]$) and early-stopping based on cross-validation performance Prechelt (1998). To limit the computational burden, If the performance

is not improved for a number of consecutive new hidden layer sizes ($maxSievePatience$), then the procedure stops and returns the current best model. For illustration, a simplified training procedure is described in Algorithm 1. The undefined ”train” function, corresponds to each neural-network’s training loop and also implicitly uses early-stopping to control the number of training epochs of each proposed architecture.

The following plot shows that algorithm 1 results in density estimates with a lower loss than their GLM counterpart, hence validating the approach.

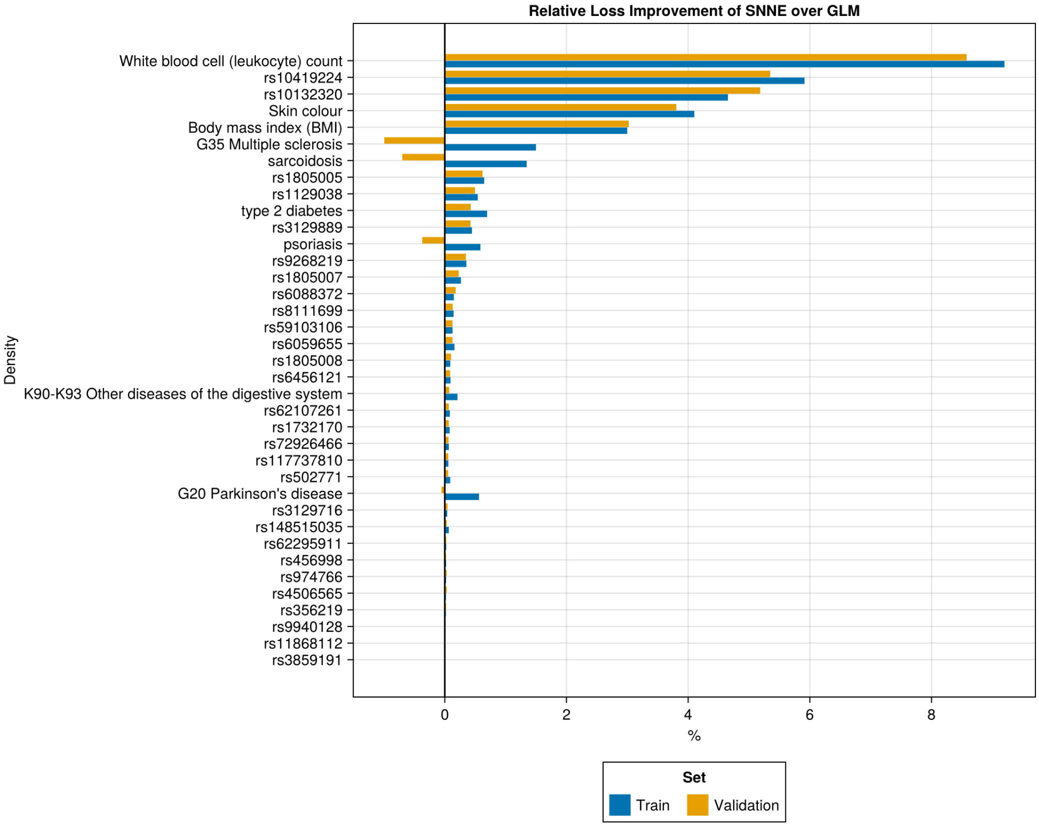

Fig. S1. Comparison of the empirical loss between the proposed Sieve Neural Network Estimator and a Generalised Linear Model baseline. For each density (y-axis), results are presented as a relative improvement of the SNNE over the GLM (x-axis). Bars facing to the right of the thick 0-line indicate an improvement while bars facing to the left indicate a deterioration of the loss. Both Train (Blue) and Validation (Yellow) set improvements are presented. These results validate the proposed density estimation strategy as an effective flexible and data-adaptive method.

All Estimands

Type	Outcome	Outcome Type	Variants	Outcome Freq	Variants Min Freq	Joint Min Freq
AIE	Body mass index	Continuous	rs62107261;rs9940128		4.2e-04	
AIE	Leukocyte count	Continuous	rs3859191;rs9268219		3.3e-03	
AIE	Multiple sclerosis	Binary	rs10419224;rs59103106	4.0e-03	3.9e-04	2.1e-06
AIE	Multiple sclerosis	Binary	rs3129889;rs62295911	4.0e-03	8.4e-05	2.1e-06
AIE	Other diseases of the digestive system	Binary	rs3129716;rs72926466	5.3e-02	3.0e-04	1.8e-05
AIE	Parkinson's disease	Binary	rs11868112;rs356219;rs6456121	6.5e-03	2.1e-03	1.8e-05
AIE	Parkinson's disease	Binary	rs1732170;rs356219;rs456998;rs8111699	6.5e-03	1.4e-03	6.2e-06
AIE	Psoriasis	Binary	rs10132320;rs974766	1.0e-02	1.0e-04	4.1e-06
AIE	Sarcoidosis (D86)	Binary	rs148515035;rs502771	2.3e-03	2.5e-05	2.1e-06
AIE	Skin colour	Count	rs1129038;rs1805008		3.6e-04	
AIE	Skin colour	Count	rs1805005;rs6059655		1.6e-04	
AIE	Skin colour	Count	rs1805007;rs6088372		3.0e-04	
AIE	Type 2 diabetes	Binary	rs117737810;rs4506565	8.8e-03	7.8e-05	2.1e-06
AIE	psoriasis	Binary	rs10132320;rs974766	1.2e-02	1.0e-04	2.1e-06
AIE	sarcoidosis	Binary	rs148515035;rs502771	2.1e-03	2.5e-05	2.1e-06
ATE	Body mass index	Continuous	rs62107261		2.2e-03	
ATE	Body mass index	Continuous	rs9940128		1.8e-01	
ATE	Leukocyte count	Continuous	rs3859191		2.2e-01	
ATE	Leukocyte count	Continuous	rs9268219		1.5e-02	
ATE	Multiple sclerosis	Binary	rs3129889	4.0e-03	2.0e-02	2.5e-04
ATE	Multiple sclerosis	Binary	rs62295911	4.0e-03	3.1e-03	2.7e-05
ATE	Other diseases of the digestive system	Binary	rs3129716	5.3e-02	2.1e-02	1.9e-03
ATE	Other diseases of the digestive system	Binary	rs72926466	5.3e-02	1.6e-02	8.4e-04
ATE	Sarcoidosis (D86)	Binary	rs148515035	2.3e-03	3.7e-04	2.1e-06
ATE	Sarcoidosis (D86)	Binary	rs502771	2.3e-03	7.5e-02	3.1e-04
ATE	Type 2 diabetes	Binary	rs117737810	8.8e-03	9.2e-04	1.0e-05
ATE	Type 2 diabetes	Binary	rs4506565	8.8e-03	1.0e-01	1.4e-03
ATE	sarcoidosis	Binary	rs148515035	2.1e-03	3.7e-04	4.1e-06
ATE	sarcoidosis	Binary	rs502771	2.1e-03	7.5e-02	3.2e-04

Table 1. The 29 estimands used across the simulation study. The "Variants Min Freq" column represents the minor genotype frequency for the variants in the estimand. When the outcome is binary, the frequency is provided as well as the "Joint Min Freq". The latter represents the minor frequency of joint (genotype, outcome).

Power of OSE

This plot is the counterpart of Figure 4 for the one-step estimator. It shows similar results.

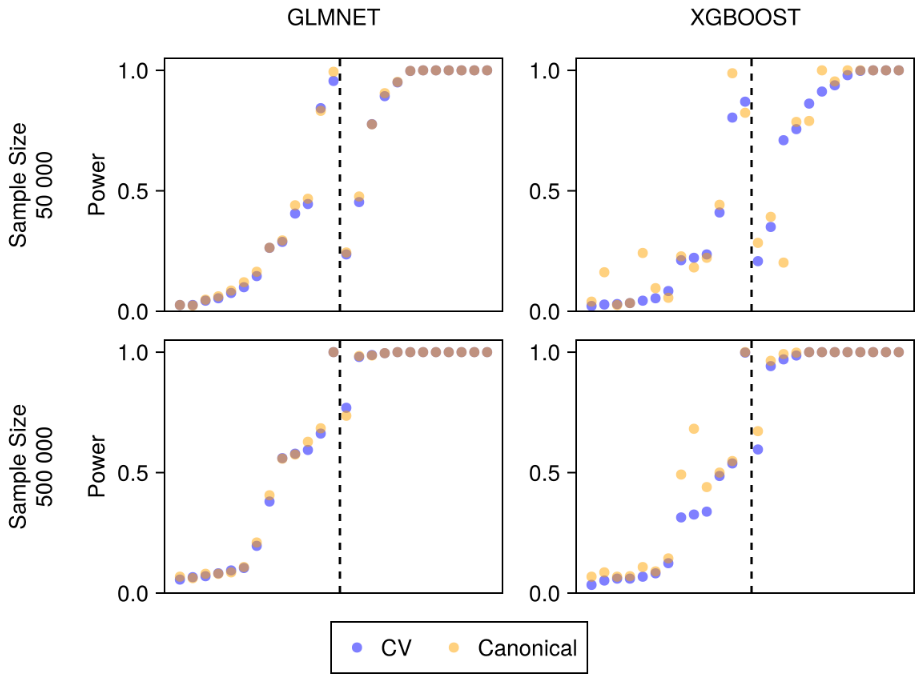

Fig. S2. Power analysis of OSE. Rows indicate sample sizes with $n = 50\,000$ (top) and $n = 500\,000$ (bottom), columns indicate the model used to fit nuisance functions with GLMNet (left) and XGBoost (right), and colour indicates the resampling scheme, *i.e.*, cross-validated (blue) and canonical (orange). Each dot corresponds to a single estimand and the dashed lines separate AIEs (left) and ATEs (right).

Analysis of Correlated Variants

In Section 3.2.2, we assumed the interacting genetic variants to be independent given the observed confounding variables. However, in population genetics, due to LD, this is not necessarily the case if one only accounts for population stratification. To illustrate this, consider the causal diagram of Figure S3A. In this model, A_1 and A_2 are in LD and cause Y. They are confounded by population stratification and LD. A putative causal variant A_0, also in LD with A_1 and A_2, could further confound the interaction effect. However, except for fine-mapping studies, A_0 is typically ignored in population genetics analyses and we do so in this simulation. The main difference with the simulation of Section 3.2.2 is that the dependence between A_1 and A_2 has to be taken into account when estimating the propensity score. More precisely, we have $P(A_1, A_2|PCs) = P(A_1|PCs) \cdot P(A_2|A_1, PCs)$. Because the LD variable does not affect Y through any other variant, the interaction effect between A_1 and A_2 is still unconfounded and can be estimated. Note however, that the ATE of either A_1 or A_2 would be confounded. In this sense, interaction studies enhance the identifiability of causal parameters in population genetics.

We introduce a dependence structure between A_1 and A_2 via the copula method. More precisely, we take as generating process

$$\begin{aligned} PC, LD &\sim \mathcal{U}(0,1) \\ dA &\sim \mathcal{N}(\mu_{dA}, \Sigma_{dA}) \\ A &= \mathbb{1}_{dA>0} \\ Y &\sim \mathcal{N}(1 + 10 \cdot A_1 - 3 \cdot A_2 \cdot A_1 \cdot PC, \sigma_Y) \end{aligned} \tag{34}$$

with $\mu_{dA} = \begin{bmatrix} PC + LD \\ PC + LD \end{bmatrix}$, $\Sigma_{dA} = \begin{bmatrix} 1 & \sigma_A \\ \sigma_A & 1 \end{bmatrix}$, $\sigma_Y = 1$ and σ_A a parameter controlling the correlation $\rho(A_1, A_2)$ between A_1 and A_2. The relation between σ_A and $\rho(A_1, A_2)$ is displayed in Figure S3B and is seen to span the $(0, 1)$ interval. This is important since genetic variants can be highly correlated, *e.g.*, $\rho(A_1, A_2) > 0.9$.

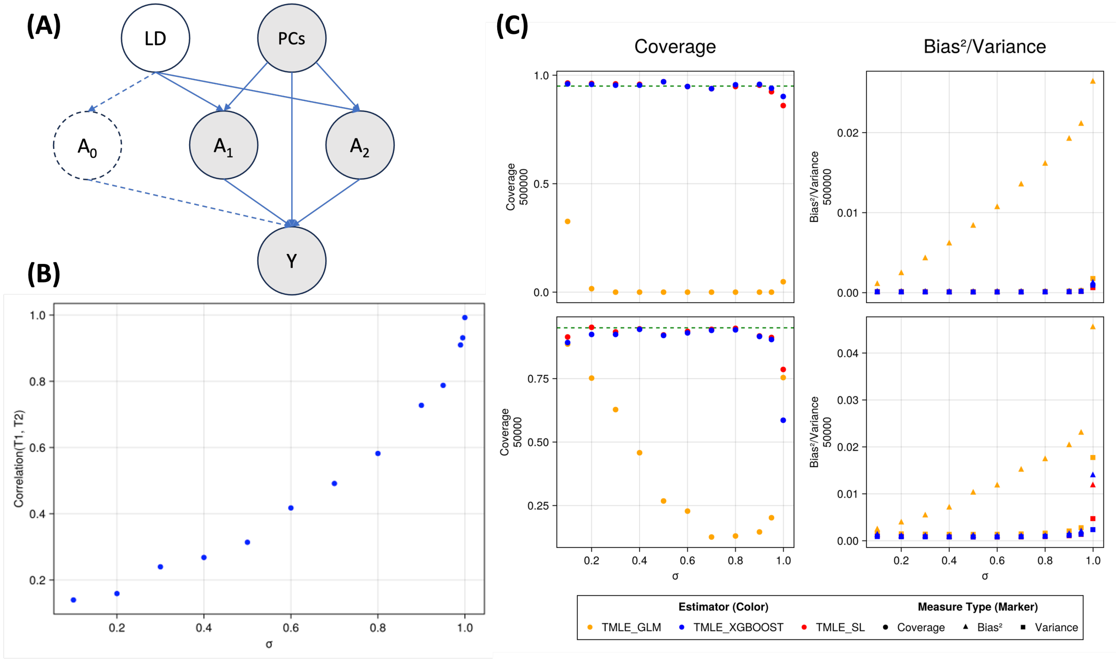

Fig. S3. Simulation results for the estimation of interaction between correlated (LD) variants. (A) Causal diagram with linkage disequilibrium. Shaded variables are observed. It is typically assumed that confounding due to population stratification can be approximated by a PC analysis, while confounding due to LD remains. **(B)** Relationship between σ_A and the induced correlation between variants, $\rho(A_1, A_2)$. Since genetic variants can exhibit very high linkage, a larger number of higher values of σ_A is sampled. **(C)** Confidence intervals obtained for each σ_A value presented in (B). The results show perfect coverage but increasing variance as the correlation between A_1 and A_2 becomes extreme.

For the same σ_A values, we estimate the interaction effect between A_1 and A_2. The true interaction effect in this model is $\text{AIE}_0 = -3 \cdot \mathbb{E}[PC] = -1.5$. In this simulation, we consistently apply the weighted TMLE estimator and investigate three different strategies to estimate the nuisance functions. The first strategy, "TMLE_GLM", uses a linear regression and logistic regression method for $\bar{Q}$ and g respectively. Importantly, the linear model does not include an interacting term to illustrate the danger of model misspecification. The second method, "TMLE_XGBOOST", uses the XGBoost learning algorithm with default parameters. Finally, the third "TMLE_SL", adaptively selects the best model (GLM or XGBoost) each trained across a grid of L_2 regularisation parameters and evaluated on a holdout set. This strategy is also known as discrete super-learning (Van der Laan and Rose, 2011). To evaluate these three strategies, we perform a bootstrap analysis ($B = 500$) for two dataset sizes ($N = 50\,000$ and $N = 500\,000$). We then report mean coverage, bias-squared and variance for each estimator (Supplementary Table 7). The results, presented in Figure S3-C, show that misspecification of the nuisance functions ("TMLE_GLM") leads to large bias and low coverage. This is in contrast to the other two methods, which provide optimal coverage for all correlations $\sigma < 0.9$. Furthermore, by adaptively selecting the best model, the super-learner also seems to provide better performance in smaller sample sizes ($N = 50\,000$) when XGBoost is prone to overfitting. The performance drop when $\sigma > 0.9$ is in agreement with the results of section 3.2 in the main text. This is because in cases where A_1 and A_2 are highly correlated, they become almost indistinguishable. This leads to practical positivity violations and instability of the propensity score estimator.

In conclusion, this simulation illustrates that in datasets similar to the UK Biobank, it is possible to estimate interactions between variants that are not too correlated ($\sigma < 0.9$). The estimation of interactions in LD blocks remains an open challenge.

Definition of Bootstrap Bias, Variance and MSE estimators.

Let n be the number of independent and identically distributed samples ($n = 50\,000$ or $500\,000$) and let B be the number of bootstrap resamples ($B = 500$). For a given estimand and estimator, each bootstrap resample yields a p-dimensional vector of estimates denoted by $\hat{\mathbf{\Psi}}_{b,n}$. For this estimate, a true value $\mathbf{\Psi}_0$ is available. We denote by $\mathbf{\Sigma}_{B,n}$ the $p \times p$-dimensional sample covariance matrix of the estimator $\hat{\mathbf{\Psi}}_n$ which is obtained from the B estimate vectors $\hat{\mathbf{\Psi}}_{b,n}$ for $b = 1, 2, \ldots, B$. We then estimate the bootstrap bias-squared, the variance and mean-squared error of our high-dimensional estimators via the following formulas:

$$\widehat{\text{Bias}}^2_{B,n} = \frac{1}{B}\sum_{b=1}^{B} ||\hat{\mathbf{\Psi}}_{b,n} - \mathbf{\Psi}_0||_2^2 \tag{35}$$

$$\widehat{\text{Var}}_{B,n} = \text{Tr}(\mathbf{\Sigma}_{B,n}) \tag{36}$$

$$\widehat{\text{MSE}}_{B,n} = \widehat{\text{Bias}}^2_{B,n} + \widehat{\text{Var}}_{B,n} \tag{37}$$

Here Tr denotes the trace of a matrix. In particular, the bootstrap variance $\widehat{\text{Var}}_{B,n}$ is the sum of the diagonal elements of the sample covariance matrix $\mathbf{\Sigma}_{B,n}$, *i.e.*, the sum of the component variance estimates.

Comparison of influence curve-based and bootstrap variance estimates.

We provide a comparison between the variance estimates obtained from the influence curve as per Equations (31) and (32), and the bootstrap variance from resampling as per Equation (36).

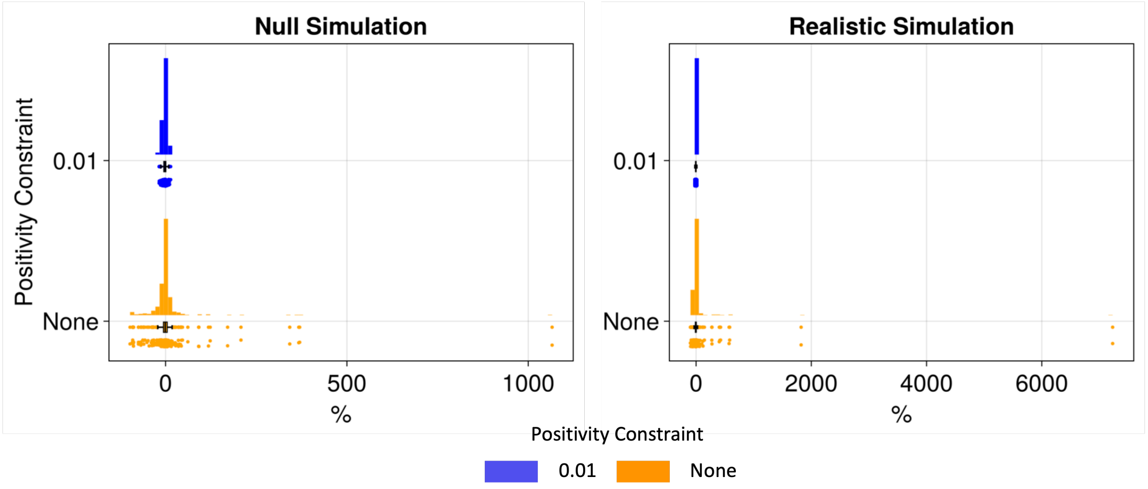

Fig. S4. Relative difference in percentages between variance estimates based on the influence curve and the bootstrap, as in Equation (36). When no positivity constraint is applied, variance estimates based on the influence curve may become extreme. When estimands are constrained at the 0.01 positivity threshold level, variance estimates get closer to each other.

SUPPLEMENT TO APPLICATIONS

PCA Analysis

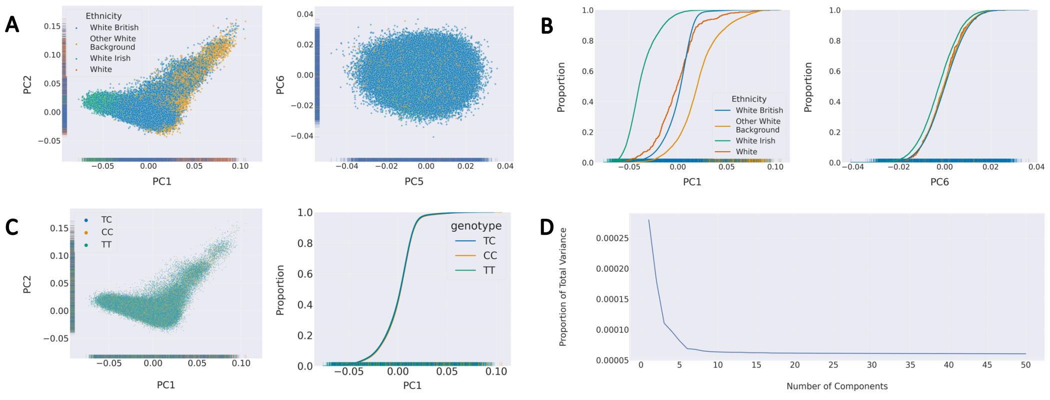

Fig. S5. Principal Component Analysis of the UK Biobank's white population (**A**) Principal component analysis labelled by ethnicity. Left: PC1 vs PC2 shows high level of population structure dependent on self-reported ethnicity. Right: PC5 vs PC6 shows a more symmetric shape suggesting that there is no ethnicity structure for PCs > 6. This is more clearly visible in (**B**) via the cumulative distribution analysis of ethnicity for PC1 and PC6. Left: The cumulative distributions of PC1 conditioned on self-reported ethnicity differ, indicating that variation in ethnicity and variation in PC1 are dependent. Right: In PC6 this separation has disappeared. (**C**) A variant-specific analysis showing that rs1421085 is not stratified in the population. The same pattern of non-stratification holds for the first 20 principal components, see Figure S6. When this is the case, principal components are not confounding the genotype-phenotype relationship. (**D**) This scree plot shows that the proportion of variance explained by each additional PC plateaus after 6 PCs, when subset on 'self-reported White' UK Biobank population, indicating that 6 PCs is sufficient to explain the population structure of this cohort.

Supplement to Figure 5: PCs for rs1421085

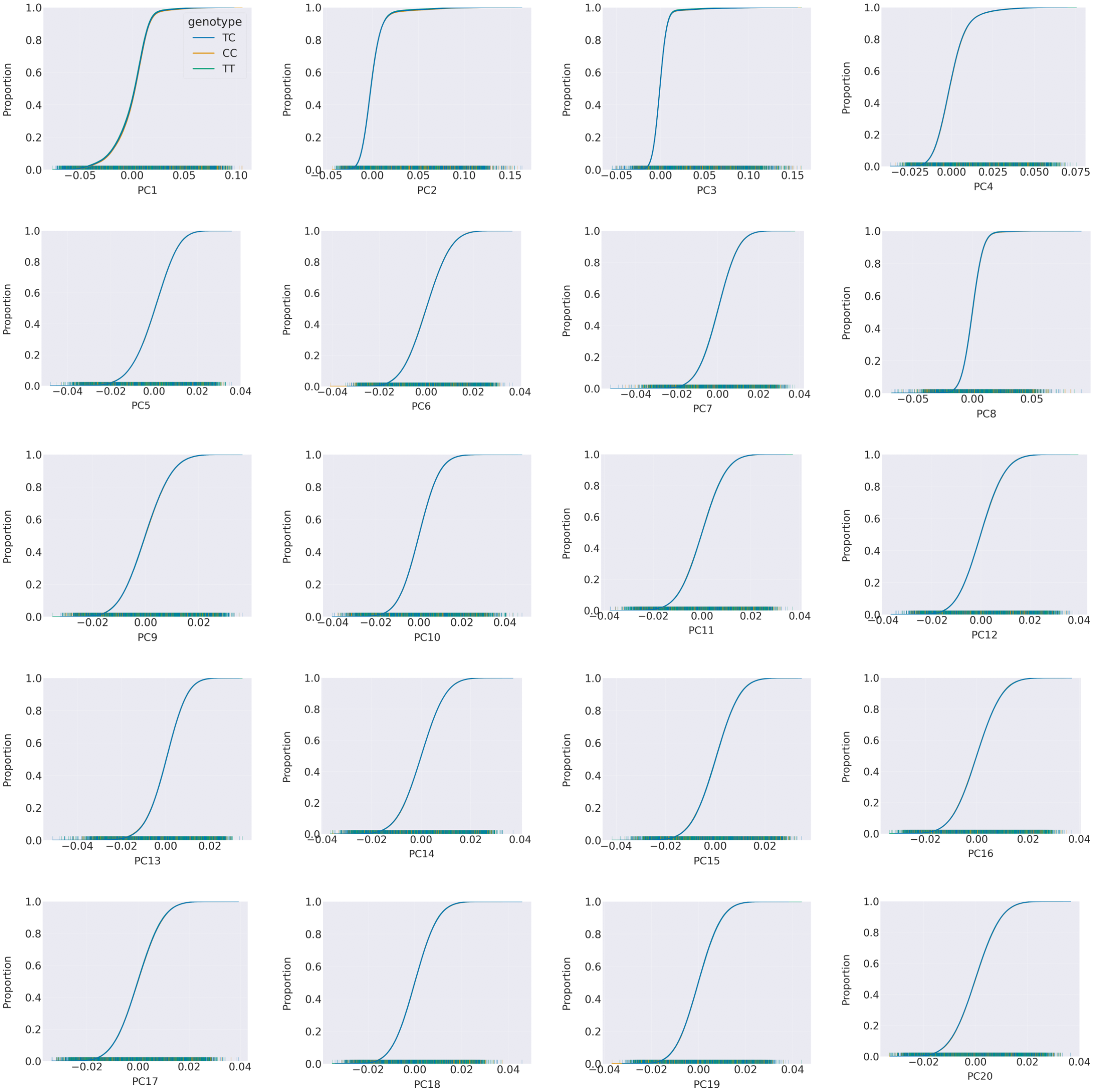

Fig. S6. Cumulative density functions of the first 20 principal components stratified by rs1421085 genotype. Population stratification plays no discernible role in the genotype distribution of the FTO variant rs1421085 in the UKB population with white ethnic background.

Supplement to Figure 5: `TarGene` results

Figure S7A provides further information on the Sieve Plateau variance corrected p-values, whereas Figure S7B shows an example Sieve Variance Plateau curve. Figure S7C illustrates the difference between the initial estimate, reported by Super Learning, and TMLE after the targeting step. Figure S7D provides a histogram of the genetic relationship matrix values on the UK Biobank population.

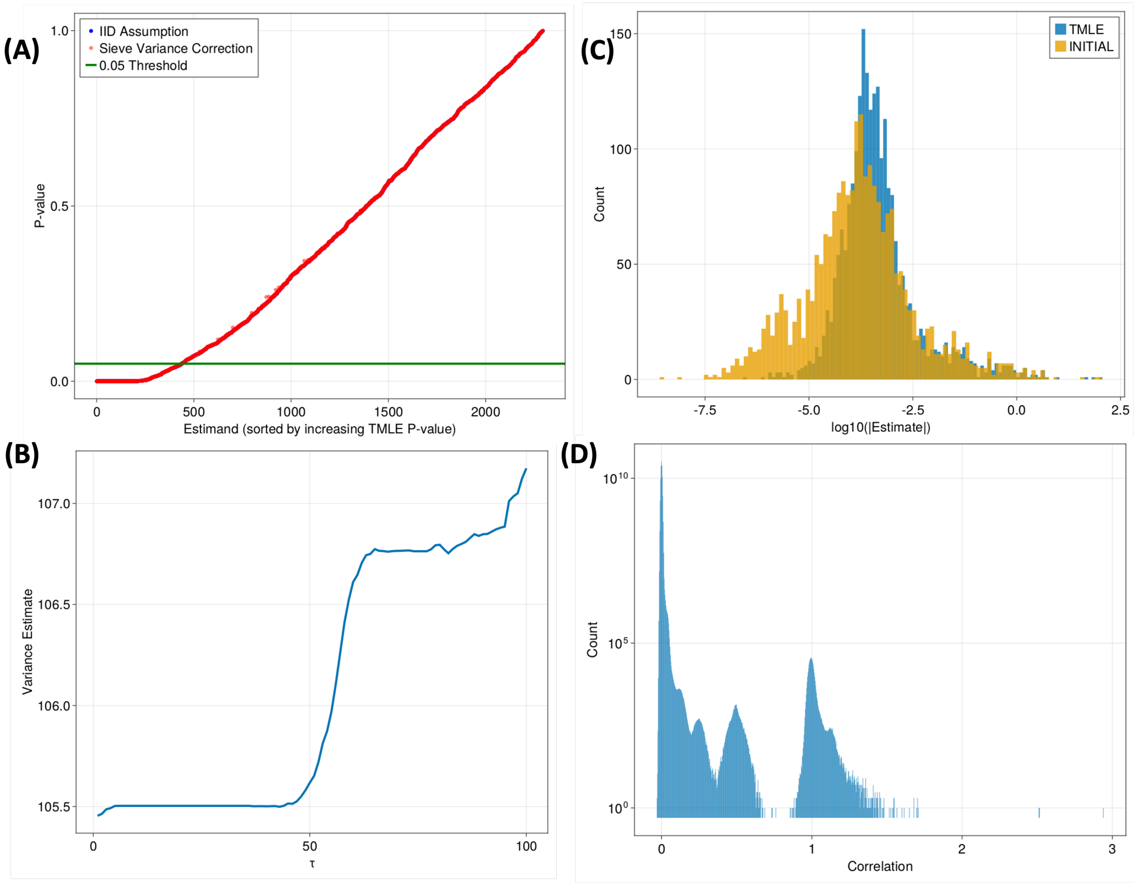

Fig. S7. Supplementary figures related to Targeted Maximum Likelihood Estimation and Sieve Variance Plateau correction. (**A**) **Sieve Variance Plateau corrected p-values.** Evidence of no significant difference across traits for rs1421085 between corrected p-values and p-values assuming individuals are independent. (**B**) **Sieve Variance Plateau curve.** A sample Sieve-Variance-Plateau curve for body mass index across 100 different thresholds. (**C**) **Difference between initial and TMLE estimates.** The initial estimate is obtained by plugin of our first Super Learning estimate for Q. In more than 78% of the cases, TMLE is driving the initial estimate towards more extreme values. This is a potential piece of evidence for the omnigenic model (Boyle et al., 2017). (**D**) **Distribution of the Genetic Relationship Matrix.** Most individuals in the UK-Biobank have low genetic similarity.

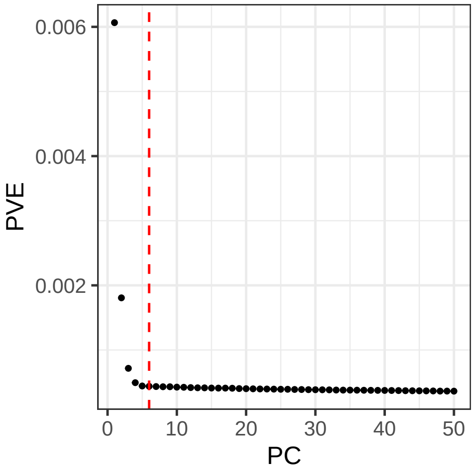

Fig. S8. All of Us (AoU) Principal Component Analysis. Principal components were computed using FlashPCA2 across all genotyped SNPs, excluding SNPs in LD with rs1421085, for 122,752 participants in the AoU cohort. The y-axis shows the proportion of variance explained (y-axis; PVE) across the first 50 principal components (x-axis; PCs), with a dashed red line at 6 PCs. The proportion of variance explained plateaus at 6 PCs, and is sufficient to capture confounding due to population stratification in the AoU cohort. This is consistent with results in UKB for the same variant.

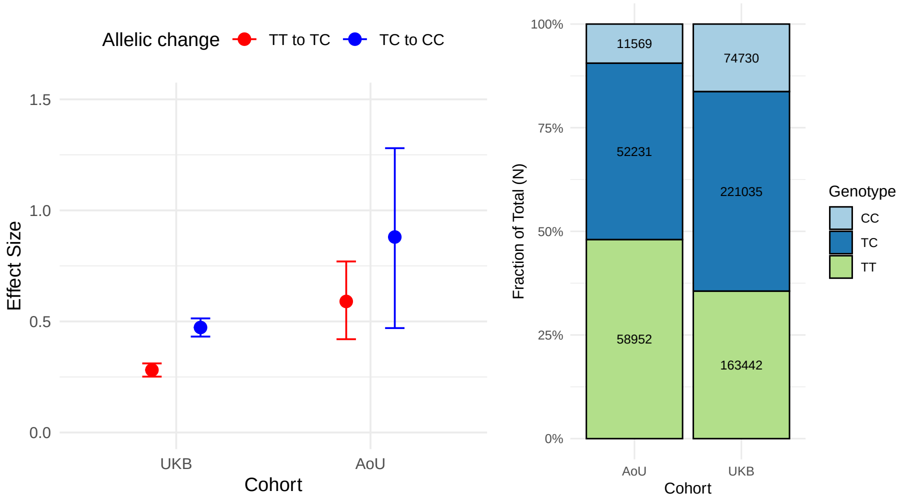

Fig. S9. All of Us (AoU) cohort compared to UKB for the effect of rs1421085 on BMI. (Left) Estimates of the individual allelic effects of rs1421085 on BMI for the UKB and AoU cohort with 95% confidence intervals. Confidence intervals do not overlap for TT-to-TC effect estimates (red), but they do overlap for TC-to-TT estimates (blue) across cohorts. A higher degree of uncertainty is found in estimates for the AoU cohort. Although a significant non-linear effect was found in the UKB for this association, this is not reproduced in the AoU cohort. This may be due to reduced power as a result of AoU's lower sample size, or due to increased variation as a result of larger environmental effects. **(Right)** Decrease in minor allele frequency (0.40 in UKB; 0.30 in AoU) and sample size (459,207 in UKB; 122,752 in AoU). Here we show a breakdown of sample sizes for each genotype across the UKB and AoU cohort, with the fraction of the cohort shown on the y-axis and the sample size for each group labelled in text.

REFERENCES

S. V. Beentjes and A. Khamseh. Higher-order interactions in statistical physics and machine learning: A model-independent solution to the inverse problem at equilibrium. *Phys. Rev. E*, 102:053314, Nov 2020. doi: 10.1103/PhysRevE.102.053314. URL https://link.aps.org/doi/10.1103/PhysRevE.102.053314.

D. Benkeser, M. Carone, M. J. V. D. Laan, and P. B. Gilbert. Doubly robust nonparametric inference on the average treatment effect. *Biometrika*, 104(4):863–880, 10 2017. ISSN 0006-3444. doi: 10.1093/biomet/asx053. URL https://doi.org/10.1093/biomet/asx053.

P. Bickel, C. Klaassen, Y. Ritov, and J. Wellner. *Efficient and Adaptive Estimation for Semiparametric Models*. Johns Hopkins series in the mathematical sciences. Springer New York, 1998. ISBN 9780387984735. URL https://books.google.co.uk/books?id=lSnTm6SC_SMC.

C. M. Bishop. Mixture density networks. 1994.

E. Boyle, L. Yi, and J. Pritchard. An expanded view of complex traits: From polygenic to omnigenic. *Cell*, 6 (169):1177–118, 2017. doi: 10.1016/j.cell.2017.05.038.

O. Canela-Xandri, K. Rawlik, and A. Tenesa. An atlas of genetic associations in UK Biobank. *Nature genetics*, 50(11):1593–1599, 2018.

X. Chen. Large sample sieve estimation of semi-nonparametric models. *Handbook of econometrics*, 6:5549–5632, 2007.

E. Elhaik. Principal component analyses (pca)-based findings in population genetic studies are highly biased and must be reevaluated. *Scientific Reports*, 12(1):14683, 2022.

S. Haykin. *Neural networks: a comprehensive foundation*. Prentice Hall PTR, 1998.

K. Hornik, M. Stinchcombe, and H. White. Multilayer feedforward networks are universal approximators. *Neural networks*, 2(5):359–366, 1989.

K. J. Lee and A. Schuler. Rieszboost: Gradient boosting for riesz regression, 2025. URL https://arxiv.org/abs/2501.04871.

P.-R. Loh, G. Tucker, B. K. Bulik-Sullivan, B. J. Vilhjálmsson, H. K. Finucane, R. M. Salem, D. I. Chasman, P. M. Ridker, B. M. Neale, B. Berger, et al. Efficient bayesian mixed-model analysis increases association power in large cohorts. *Nature genetics*, 47(3):284–290, 2015.

J. Mbatchou, L. Barnard, J. Backman, A. Marcketta, J. A. Kosmicki, A. Ziyatdinov, C. Benner, C. O'Dushlaine, M. Barber, B. Boutkov, et al. Computationally efficient whole-genome regression for quantitative and binary traits. *Nature genetics*, 53(7):1097–1103, 2021.

J. Pfanzagl and W. Wefelmeyer. Contributions to a General Asymptotic Statistical Theory. *Statistics & Risk Modeling*, 3(3-4):379–388, April 1985. doi: 10.1524/strm.1985.3.34.37. URL https://ideas.repec.org/a/bpj/strimo/v3y1985i3-4p379-388n13.html.

K. E. Porter, S. Gruber, M. J. van der Laan, and J. S. Sekhon. The relative performance of targeted maximum likelihood estimators. *The International Journal of Biostatistics*, 7(1):0000102202155746791308, 2011.

L. Prechelt. Automatic early stopping using cross validation: quantifying the criteria. *Neural networks*, 11(4): 761–767, 1998.

A. L. Price, N. J. Patterson, R. M. Plenge, M. E. Weinblatt, N. A. Shadick, and D. Reich. Principal components analysis corrects for stratification in genome-wide association studies. *Nature genetics*, 38(8):904–909, 2006.

J. M. Robins, A. Rotnitzky, and L. P. Zhao. Estimation of regression coefficients when some regressors are not always observed. *Journal of the American Statistical Association*, 89(427):846–866, 1994. ISSN 01621459, 1537274X. URL http://www.jstor.org/stable/2290910.

D. B. Rubin. Estimating causal effects of treatments in randomized and nonrandomized studies. *Journal of Educational Psychology*, 66(5):688–701, 1974. doi: https://doi.org/10.1037/h0037350.

O. Sofrygin and M. J. Van der Laan. Semi-parametric estimation and inference for the mean outcome of the single time-point intervention in a causally connected population. *Journal of Causal Inference*, 5(1), 2017.

M. Van der Laan and J. Robins. *Unified Methods for Censored Longitudinal Data and Causality*. Springer Series in Statistics. Springer, 2003. ISBN 9780387955568.

M. J. Van der Laan and S. Rose. *Targeted Learning: Causal inference for observational and experimental data*. Springer Series in Statistics. Springer, New York, 2011. ISBN 978-1-4419-9781-4. URL https://doi.org/10.1007/978-1-4419-9782-1.

M. J. Van der Laan and S. Rose. *Targeted Learning in Data Science: Causal inference for complex longitudinal studies*. Springer Series in Statistics. Springer, Cham, 2018. ISBN 978-3-319-65303-7; 978-3-319-65304-4. URL https://doi.org/10.1007/978-3-319-65304-4.

M. J. Van der Laan and D. Rubin. Targeted Maximum Likelihood Learning. *The International Journal of Biostatistics*, 2(1), 2006.

J. Yang, S. H. Lee, M. E. Goddard, and P. M. Visscher. Gcta: a tool for genome-wide complex trait analysis. *The American Journal of Human Genetics*, 88(1):76–82, 2011.
