# Supplementary material for: Semiparametric efficient estimation of small genetic effects in large-scale population cohorts
Source: Biostatistics. 2025 Sep 30;26(1):kxaf030. doi: 10.1093/biostatistics/kxaf030 (PMC12479317; doi:10.1093/biostatistics/kxaf030)
Supplement: kxaf030_Supplementary_Data [file kxaf030_supplementary_data.zip › biosts-25031-File004.zip!/figs_supplement/AllofUs_FTO_ScreePlot.pdf]

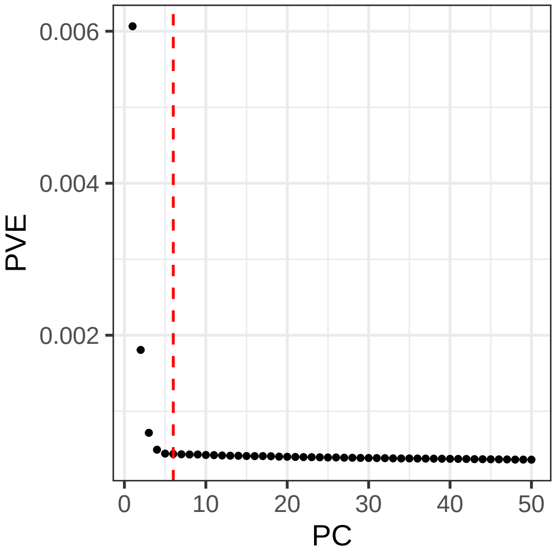
0.006
0.004
0.002
PVE
0
10
20
30
40
50
PC
