# Supplementary material for: Semiparametric efficient estimation of small genetic effects in large-scale population cohorts
Source: Biostatistics. 2025 Sep 30;26(1):kxaf030. doi: 10.1093/biostatistics/kxaf030 (PMC12479317; doi:10.1093/biostatistics/kxaf030)
Supplement: kxaf030_Supplementary_Data [file kxaf030_supplementary_data.zip › biosts-25031-File004.zip!/figs_supplement/Sample_sizes_by_genotype_AoU_UKB.pdf]

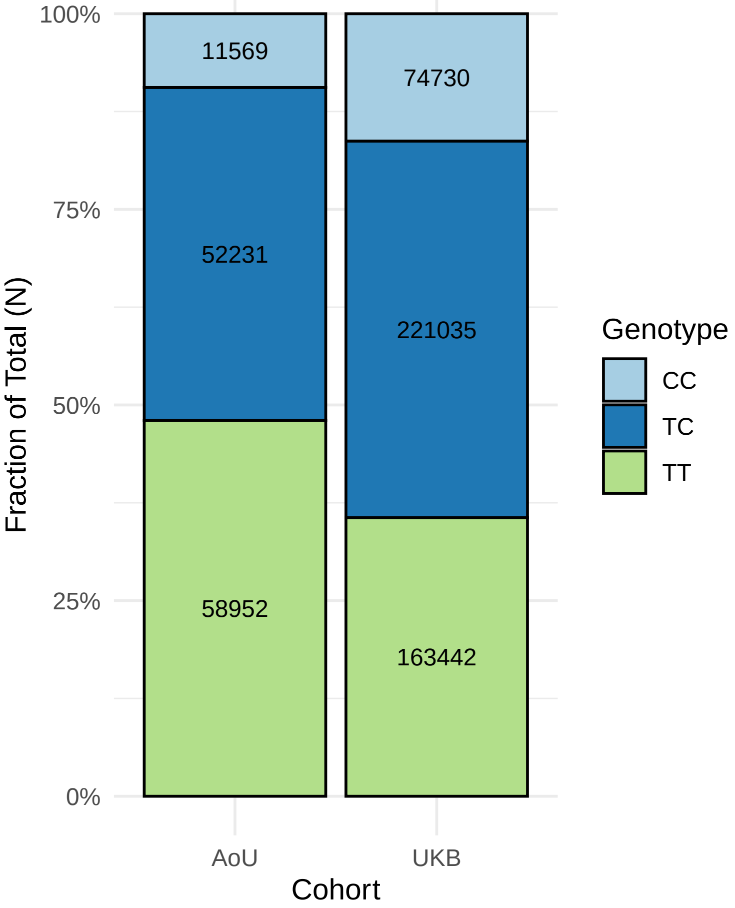

Fraction of Total (N)
100%
75%
50%
25%
0%
11569
52231
58952
74730
221035
163442
AoU
UKB
Cohort
Genotype
CC
TC
TT
