# Supplementary material for: Semiparametric efficient estimation of small genetic effects in large-scale population cohorts
Source: Biostatistics. 2025 Sep 30;26(1):kxaf030. doi: 10.1093/biostatistics/kxaf030 (PMC12479317; doi:10.1093/biostatistics/kxaf030)
Supplement: kxaf030_Supplementary_Data [file kxaf030_supplementary_data.zip › biosts-25031-File004.zip!/figs_supplement/PCA_picture.pdf]

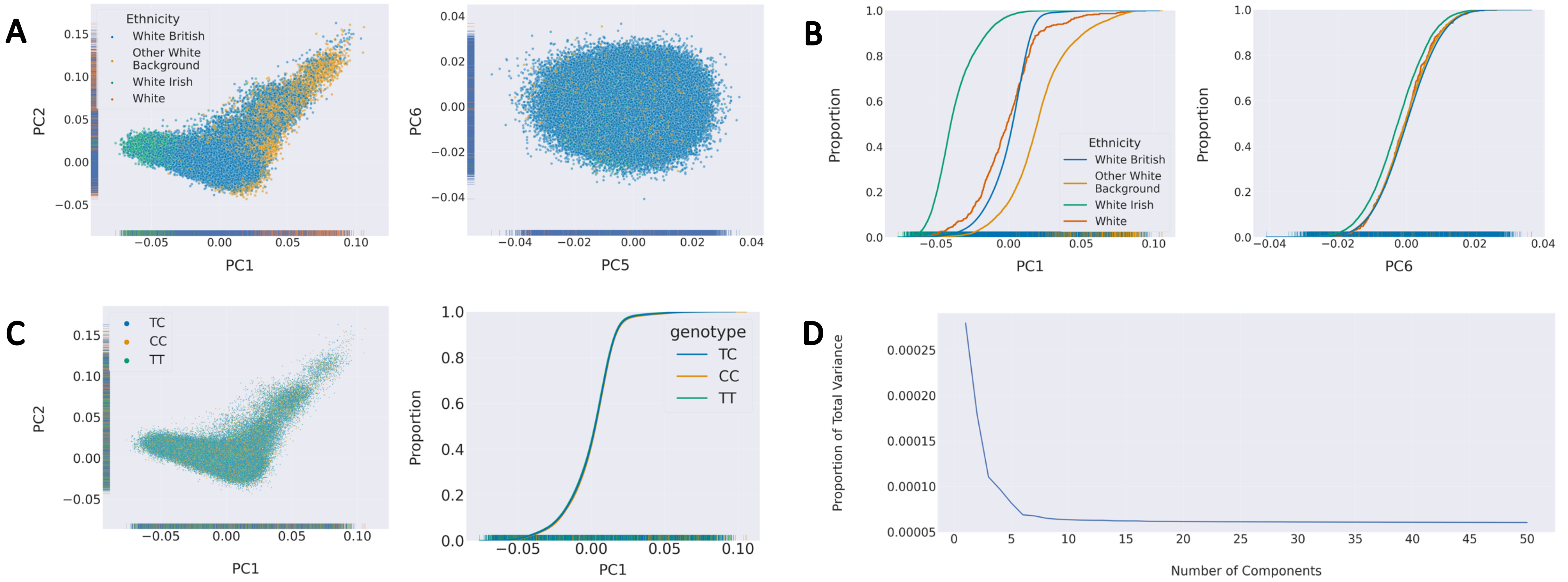
A
Ethnicity
White British
Other White Background
White Irish
White
PC1
PC2
PC5
PC6
B
Proportion
Ethnicity
White British
Other White Background
White Irish
White
PC1
PC6
C
TC
CC
TT
PC1
PC2
genotype
TC
CC
TT
Proportion
PC1
D
Proportion of Total Variance
Number of Components
